# Citrate‐Coated Iron Oxide Nanoparticles Facilitate Endothelialization of Left Ventricular Assist Device Impeller for Improved Antithrombogenicity

**DOI:** 10.1002/advs.202408976

**Published:** 2024-12-20

**Authors:** Jasper L. Haritz, Michael Pflaum, Hans J. Güntner, Katherina Katsirntaki, Jan Hegermann, Felix Hehnen, Michael Lommel, Ulrich Kertzscher, Jutta Arens, Axel Haverich, Arjang Ruhparwar, Bettina Wiegmann

**Affiliations:** ^1^ Department of Cardiothoracic Transplantation and Vascular Surgery Hannover Medical School Carl‐Neuberg‐Str. 1 30625 Hannover Germany; ^2^ Lower Saxony Center for Biomedical Engineering Implant Research and Development Stadtfelddamm 34 30625 Hannover Germany; ^3^ German Center for Lung Research BREATH Hannover Medical School Carl‐Neuberg‐Str. 1 30625 Hannover Germany; ^4^ Research Core Unit Electron Microscopy and Institute of Functional and Applied Anatomy Hannover Medical School Carl‐Neuberg‐Str. 1 30625 Hannover Germany; ^5^ Biofluid Mechanics Laboratory Institute of Computer‐assisted Cardiovascular Medicine Charité – Universitätsmedizin Berlin 13353 Berlin Germany; ^6^ Charité –Universitätsmedizin Berlin corporate member of Freie Universität Berlin and Humboldt‐Universität zu Berlin Charitéplatz 1 10117 Berlin Germany; ^7^ Engineering Organ Support Technologies Group Department of Biomechanical Engineering Faculty of Engineering Technology University of Twente Enschede NB 7522 Netherlands; ^8^ Member of the DFG‐SPP2014 30625 Hannover Germany

**Keywords:** endothelialization, heart failure, hemocompatibility, LVAD, magnetic nanoparticles

## Abstract

Although left ventricular assist devices (LVADs) are an alternative to heart transplantation, their artificial surfaces often lead to serious thrombotic complications requiring high‐risk device replacement. Coating blood‐contacting surfaces with antithrombogenic endothelial cells is considered an effective strategy for preventing thrombus formation. However, this concept has not yet been successfully implemented in LVADs, as severe cell loss is to be expected, especially on the impeller surface with high prothrombogenic supraphysiological shear stress. This study presents a strategy that exploits the magnetic attraction of the impeller on ECs loaded with iron oxide nanoparticles (IONPs) to minimize shear stress‐induced cell detachment from the rotating magnetic impeller while ensuring antithrombogenic EC adhesion, especially as a bridge until they formed their adhesion‐promoting matrix. In contrast to polyvinylpyrrolidone (PVP)‐coated IONPs, more efficient and safer cell loading is achieved with sodium citrate (Cit)‐stabilized IONPs, where incubation with 6.6 µg iron mL‐1 Cit‐IONPs for 24 h resulting in an average internalization of 23 pg iron per cell. Internalization of Cit‐IONP significantly improved cell attraction to the highly magnetic impeller surface without affecting cell viability or antithrombogenic function. This protocol is key for the development of a biohybrid LVAD impeller that can prevent life‐threatening thrombosis and hemorrhage in a future clinical application.

## Graphical abstract

Pro‐thrombogenic surfaces of left ventricular assist devices (LVAD) require anticoagulant therapy, which leads to bleeding and thrombi in an unbalanced state. To avoid this, an antithrombogenic endothelial cell (EC) layer is applied. The ECs are loaded with citrate‐coated iron oxide nanoparticles (Cit‐IONP) to withstand the high shear stresses in clinical use due to the magnetic attraction of the LVAD impeller.

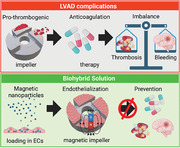

## 1 Introduction

According to the World Health Organization (WHO), heart failure (HF) is the leading cause of death worldwide, and therefore is considered the non‐communicable epidemic of the 21^st^ century.^[^ ^1^ ^]^ Between 1990 and 2017, the number of affected patients almost doubled to more than 64 million worldwide,^[^ ^2^ ^]^ whereas the number of deceased patients has recently been plateauing at around 9 million per year.^[^ ^3^, ^4^ ^]^ This increasing prevalence is due to demographic changes in many industrialized countries as well as to the constant improvements in diagnostics and the ongoing development of evidence‐based therapies.^[^ ^5^, ^6^, ^7^ ^]^ Nevertheless, patients with advanced heart failure (AdHF), currently 2–15%, are refractory to purely conventional therapy.^[^ ^8^, ^9^, ^10^ ^]^ In these cases, the last resort of modern medicine is heart transplantation (HTx) which is considered the gold standard with a median survival time of 12 years.^[^ ^11^ ^]^ Unfortunately, the number of patients who can be transplanted is limited due to the increasing shortage of donor organs.^[^ ^12^ ^]^

However, with the introduction of left ventricular assist devices (LVADs), valid alternative therapeutic care strategies for patients with AdHF have been developed: Patients can be bridged either to HTx or, less frequently, to recovery and explantation. Commonly, LVAD implantation is even performed instead of HTx as a valid alternative as so‐called “final destination therapy”.^[^ ^13^, ^14^, ^15^ ^]^ Technological progress has made it possible for LVADs to run almost wear‐free today and to be implanted much more easily using minimally invasive surgical techniques.^[^ ^16^ ^]^ Today, short‐term clinical results are comparable to those of transplanted patients.^[^ ^17^ ^]^ However, since LVAD implantation is performed as “final destination therapy” in more than 70% of patients,^[^ ^18^ ^]^ quality of life and long‐term survival are of crucial importance and still lag significantly behind HTx.^[^ ^19^ ^]^ Limiting factors result from the inevitable contact of the circulating blood with the artificial, pro‐thrombogenic LVAD surfaces.^[^ ^20^, ^21^ ^]^ While high shear rates generated in the LVAD activate the platelets,^[^ ^22^ ^]^ contact activation promotes blood coagulation with accompanying protein adsorption, consecutive platelet adhesion^[^ ^20^ ^]^ and activation of the complement system.^[^ ^21^ ^]^ Therefore, strictly adapted anticoagulation therapy of the patient is indispensable to prevent thrombogenesis within the LVAD, which otherwise lead to its (partial) occlusion and necessitate surgical and risky device replacement.^[^ ^18^ ^]^ It can also lead to serious and sometimes fatal organ infarctions (e.g. stroke).^[^ ^18^, ^23^ ^]^ However, anticoagulation also carries the risk of uncontrollable, sometimes fatal bleeding (e.g., intracerebral hemorrhage). Therefore, patients often die prematurely from LVAD‐related causes.^[^ ^17^ ^]^

A promising approach to avoid such complications comes from the field of tissue engineering and is the endothelialization of artificial blood‐contacting surfaces.^[^ ^24^ ^]^ In the human vascular system, the endothelial monolayer (EML) acts as physiological, antithrombogenic and anti‐inflammatory barrier between blood components and the pro‐thrombogenic matrix of the vessel wall. It consists of endothelial cells (EC) that resist the high shear forces of the blood flow by forming strong adhesion complexes between the individual cells (e.g., via VE‐cadherin), but also with the underlying extracellular matrix of the vessel wall, the basement membrane.^[^ ^25^ ^]^ Therefore, the basic hypothesis of this project is that a functionally confluent, antithrombogenic EML on the blood‐contacting LVAD surfaces can significantly prevent the aforementioned adverse reactions by maintaining physiological hemostasis. This would eliminate the need for anticoagulant medication and significantly improve both quality of life and long‐term survival. These advantages of endothelialization have already been shown to be effective in other biohybrid approaches.^[^ ^26^, ^27^, ^28^ ^]^ In order to be able to utilize these physiological properties of the ECs on the LVAD surfaces in long term, the resistance of the monolayer to tangential supraphysiological shear forces, which occur in particular on the rotating impeller, must be guaranteed. With regard to the translational capability of this approach, however, the challenge is that this cellular flow resistance must be immediately available in order to be used effectively, especially in the often‐urgent LVAD implantations. Since the maturation and built up of a protective matrix and the necessary intercellular connections need some time to be established, we have developed a strategy that facilitates immediate EC adhesion to the impeller during this critical time frame. Here, we took advantage of the highly magnetic impeller surface and devised a protocol for the endothelial internalization of sterically (polyvinylpyrrolidone) or electrostatically (sodium citrate) stabilized iron oxide nanoparticles (IONPs), so that both the immediate adhesion and the efficiency of endothelialization were significantly improved due to the magnetic interactions. Thus, this study is the first to elaborated a clinically relevant concept for bridging this critical time window between endothelialization and matrix formation.

## 2 Experimental Section

### 2.1 Isolation and Cultivation of Endothelial Cells (EC)

Human umbilical cord blood‐derived colony‐forming endothelial cells (hCB‐ECFCs) were isolated and cultured as previously described.^[^ ^27^ ^]^ Briefly, cord blood was collected immediately after birth in tubes containing heparin (25 I.U. mL^−1^) with the consent of the mother and in accordance with local ethical regulations (Institutional Review Board No. 1303–2012). After a 1:1 dilution with phosphate‐buffered saline (PBS) containing 2 mm ethylenediaminetetraacetic acid (EDTA), the blood was filtered through 40 µm mesh cell sieves and subjected to density gradient centrifugation using BioColl (Biochrome AG, Germany). Mononuclear cells were collected from interphase and seeded on culture plastic (Nunc delta treated, Thermo) in Endothelial Growth Medium (EGM‐2, Lonza) supplemented with an additional 10% fetal bovine serum (FBS). Cells were cultured in a humid atmosphere at 37°C and 5% CO_2_. After initial colony formation, the medium was replaced with normal EGM‐2 (2% FBS). Fresh EGM‐2 was added every second day and cells were subcultured with trypsin/EDTA (Bio & Sell) and DMEM (Gibco) with 10% FBS when 80% confluency was reached. Sequential passaging for hCB‐ECFC expansion was performed in 175 cm^2^ flasks, and cells were reseeded at 8 × 10^3^ cm^−2^. The hCB‐ECFC population was observed using an Olympus CKX53 microscope, and cell numbers were determined using CASY Cell Counter and Analyzer (OMNI Life Science). For simplicity, hCB‐ECFCs are referred to as ECs in this manuscript. For cryopreservation between experiments, ECs were suspended in 10% dimethylsulphoxide (DMSO) in FBS, cooled at a rate of −1 °C min^−1^ and stored at −140 °C. The experiments were performed with ECs in the eighth passage.

### 2.2 Generation of Iron Oxide Nanoparticles (IONPs)

IONP synthesis was performed by pulsed laser ablation in liquid (PLAL) at the Austrian Institute of Technology (AIT), Vienna, according to standardized and published protocols.^[^ ^29^ ^]^ Briefly, iron plates (purity 99.9%) were placed in quartz vials filled with 1 mL ultrapure water and treated with ultrashort laser pulses of 100 µJ at a rate of 5 kHz (TruMicro Series 5000, Trumpf). The obtained particles were ultracentrifuged and dispersed in water containing either 1 mM of the stabilizers sodium citrate (Cit) or polyvinylpyrrolidone (PVP). The zeta potential of the uncoated IONPs in aqueous dispersion was determined to be ‐24 mV. The stock dispersions contained 660 µg Fe mL^−1^ (Cit‐IONPs) and 485 µg Fe mL^−1^ (PVP‐IONPs). To confirm the targeted nanoparticle size, transmission electron microscopy (TEM) was performed using the Morgagni TEM (FEI, Eindhoven, NL).

### 2.3 Analysis of Different IONP Loading Protocols

Basically, confluent EC monolayers were cultured for 6 or 24 h with EGM‐2 containing Cit‐IONPs or PVP‐IONPs at different concentrations, where a 1:10 dilution of the stock dispersion in EGM‐2 was defined as high dose (HD) and a 1:100 dilution as low dose (LD). Between the different experimental setups, the volume of IONP dispersion per culture vessel area was kept constant.

#### 2.3.1 Morphological Evaluation and Qualitative Intracellular IONP Detection

After the respective IONP loading protocols, the ECs were washed with PBS to assess, on the one hand, the endothelial morphology by phase contrast using the CKX53 microscope and the SC50 camera (Olympus). On the other hand, to qualitatively assess IONP internalization, intracellular ferric ions were visualized by staining with Prussian blue. For this purpose, ECs were fixed with 100% methanol for 10 min and stained with a 1:1 mixture of hydrochloric acid (1%) and potassium ferricyanide (II) (10%, Merck) for 30 min in the dark. The subsequent washing with ultrapure water was followed by a 20‐min counterstaining with nuclear fast red aluminum sulfate solution (0.1%, Carl Roth) and two further washing steps with water. The color images of the stained samples were taken with the above‐mentioned microscope and camera.

#### 2.3.2 Assessment of Cytotoxicity‐Induced Apoptosis

The cytotoxic effect of the different IONP incubation protocols on the ECs was determined by analyzing the respective apoptosis rate using the Annexin‐V‐FITC kit (Miltenyi Biotec) according to the manufacturer's instructions. For this purpose, the ECs were detached from the culture plastic after completion of the incubation protocols, counted, and resuspended in 100 µL of the kit binding buffer. 10 µL of Annexin V‐FITC was added and the cells incubated in the dark for 10 min. Washing was repeated and cells were resuspended in 500 µL of FACS buffer (0.5% bovine serum albumin/0.02% EDTA/PBS). Immediately prior to flow cytometric analysis using MACSQuant Analyzer 10 (Miltenyi Biotec), 5 µL of propidium iodide (PI) was added to the samples. Flow cytometric data were then analyzed using FlowLogic software version 700.0A (Inivai Technologies, Mentone, Australia).

#### 2.3.3 Investigation of the Reciprocal Influence of ECs with IONP Loading Under Long‐Term Cultivation

These analyses were performed using the two most promising IONP loading protocols based on the preceding studies, i.e., ECs were loaded with either low‐dose Cit‐IONPs or high‐dose PVP‐IONPs for 24 h before being repeatedly detached for 4 cell passages and seeded into new EGM‐2 every third day. Here, possible influences on cell viability, as well as cell proliferation and the resulting cell number were quantified using the CASY cell counter. In addition, the effect of cell passage on intracellular iron content was qualitatively assessed using the Prussian blue staining described in 2.3.1.

### 2.4 First Proof‐of‐Concept for Optimal Cit‐IONP‐Loaded ECs

From the results of the previous analyses 2.3.1. to 2.3.3, the optimal IONP loading protocol was selected (Cit‐IONPs in low dosage, 24 h) and used for the subsequent analyses.

#### 2.4.1 Shoul Electron Microscopy (TEM) for Intracellular IONP Detection

ECs were grown to confluence in 75 cm^2^ flasks, exposed to the selected loading protocol, and prepared for TEM imaging as previously described to confirm the actual internalization of Cit‐IONP.^[^ ^30^ ^]^ After fixation in 150 mM HEPES containing 1.5% formaldehyde and 1.5% glutaraldehyde (pH 7.35), samples were immobilized in 2% agarose and incubated for 2 h in an aqueous solution of 1% OsO_4_ and 1.5% hexacyanoferrate II. After a subsequent washing step with water, the samples were stored overnight at 4 °C in 1% uranyl acetate, followed by another washing step. The samples were then dehydrated in acetone and embedded in Epon to make ultra‐thin sections (60 nm). These were mounted on Formvar‐coated copper grids and post‐stained with uranyl acetate and lead citrate and imaged in a Morgagni TEM (FEI). Images were taken with a side‐mounted CCD camera (Veleta).

#### 2.4.2 Quantification of Intracellular Iron Content after Cit‐IONP Loading

Using inductively coupled plasma optical emission spectrometry (ICP‐OES), the iron content of Cit‐IONP‐ECs was quantified by an independent analytical laboratory (Wessling GmbH, Hannover). For this purpose, the confluent Cit‐IONP‐loaded EML was first washed twice with PBS, trypsinized, and resuspended in EGM‐2 and its number was determined with the CASY system. Together with the reference samples (non‐loaded ECs and blank EGM‐2), the samples were further processed in the analytical laboratory by diluting them to 25 mL, adding 8 mL of aqua regia (3:1 nitric acid/hydrochloric acid) and performing microwave digestion. It was then made up to a final volume of 50 mL with ultrapure water. Final analysis was performed using the 720‐ES ICP‐OE spectrometer (Agilent Inc.), and the iron content of the Cit‐IONP‐ECs was calculated considering the cell numbers and background iron of the references.

#### 2.4.3 Application of Cit‐IONP‐ECs on Magnetized TiN‐Coated Surfaces

To provide initial proof of concept, the magnetic titanium surface of an LVAD impeller was mimicked by flat, rectangular titanium plates coated with titanium nitride (TiN) (Goodfellow GmbH, Hamburg and CeWOTec GmbH, Chemnitz, Germany). After sterilization, plates were placed in silicone molds in the center of the cell culture dishes, under which permanent magnets were glued to create a magnetic gradient in flux density (0 – 200 mT, from right to left side of plate) on each TiN plate. Cit‐IONP‐loaded ECs were seeded onto the TiN plates with a concentration of 1×10^5^ ECs mL^−1^ in EGM‐2. For real‐time monitoring of cell seeding success through the opaque plate, EC was stained with 25 µM CellTracker Red CMPTX (Thermo, Germany) in serum‐free DMEM for 30 min directly before seeding. Cultivation was performed for two days at 37° C and 5% CO_2_ on a tilting table, with EGM‐2 renewed after 4 and 24 h. Afterwards, samples were stained with 0.2 µM Calcein AM (Thermo, Germany) for 30 min and images were captured using a SteREO Discovery.V8 microscope and an AxioCam ICm1 (Zeiss, Jena, Germany). The ROIs were selected from the highest and lowest flux density areas on the TiN plate and were about 2.5 cm apart.

#### 2.4.4 Evaluation of the Expression Profile of Inflammation‐ and Coagulation‐Associated Genes of IONP‐ECs on TiN

Gene expression analysis of the pro‐inflammatory genes endothelial‐leukocyte adhesion molecule 1 (ELAM‐1), vascular cell adhesion molecule 1 (VCAM‐1) and intercellular adhesion molecule 1 (ICAM‐1) as well as the anti‐thrombogenic thrombomodulin (TM) of IONP‐loaded and non‐loaded ECs cultured on either tissue culture plastic (TCP) or TiN was performed. In addition, a portion of ECs was stimulated with TNFα (10 ng mL^−1^ for 6h) and served as a reference for the activated endothelial phenotype. Next, RNA was extracted from the respective ECs using the NuceloSpin II kit (Machery‐Nagel) and transcribed into cDNA using random hexamer primers and the RevertAid H Minus First Strand cDNA Synthesis Kit (Fermentas). Subsequently, qRT‐PCR was performed with SYBR Green Mix (Abgene, Thermo Fisher) and Lightcycler (Roche), using the housekeeping gene β‐actin for normalization. Expression values were calculated using the 2^−∆Ct^ method. Detailed information on the cycling programs and the specific primers can be found in Table S1 (Supporting Information).

### 2.5 Deployment of IONP‐Loaded ECs on the LVAD Impeller

The HVAD (HeartWare, Medtronic), which is clinically established and implanted many times, was used as the LVAD model for the following experiments. The impeller was removed from the LVAD housing and sterilized in an autoclave. Before use for the respective experiments, the magnetic properties of the individual impellers were examined with the teslameter FM 302 using the transversal probe AS‐NTP 0.6 (Projekt Elektronik GmbH, Berlin).

#### 2.5.1 Investigation of Static Impeller Endothelialization

Static colonization of the impeller was performed for 48 h in a 100 mL glass beaker filled with 20 mL EGM‐2 containing 2 × 10^6^ Cit‐IONP‐loaded or non‐loaded ECs, which served as a reference. The cell medium was renewed after 4 and 24 h. The assessment of the respective endothelialization efficiency was performed by viability staining using calcein and consecutive fluorescence microscopy. In order to view the complete surface of one impeller blade, two images were stitched. Quantification of cell numbers in three selected ROIs on two arbitrarily chosen blades was performed by manual counting using open‐source software ImageJ (National Institutes of Health, USA). These three ROIs on the impeller blade surface were selected, according to their location in areas with different magnetic field strength and proximity to distinct profile changes in the impeller topography (see Figure 4a). ROI I was located in the middle of the blade close to the inner border of the bearing area, with a field strength of 490 mT. ROI II was located close to the end of the wider side of the blade, with 400 mT field strength and ROI III with a field strength of 40 mT was located in the bridge region between the bearing region and the pressure relief edge.

Further analyses included cell lysis for subsequent gene expression analyses (see 2.4.4) and immunofluorescence detection of VE‐cadherin and collagen IV. For this purpose, the EMLs on the impeller were washed with PBS and fixed with 4% PFA for 5 min. Afterward, the ECs were permeabilized and blocked with 0.25% Triton X‐100 in Tris‐buffered saline containing 0.5% donkey serum for 10 min. Subsequently, these samples were rinsed and incubated with 2% bovine serum albumin (BSA, Roth) for 30 min. After several PBS wash steps, primary antibodies (see supplementary methods) were applied in 1% BSA/PBS for 1 h before incubation with fluorochrome‐labeled secondary donkey antibodies and Hoechst33342 nuclear dye (2 µg mL^−1^) for 30 min in the dark followed. After three washing steps using PBS, fluorescence microscopy was performed using the AxioVert A1 (Zeiss) and a compatible camera (AxioCam ICm1, Zeiss). Isotype‐matched antibodies were used for control staining of ECs that were statically cultured on TCP in parallel.

#### 2.5.2 Hemocompatibility Analysis by Platelet Adhesion Assay

The quantitative platelet adhesion test was used to investigate whether a functionally confluent EML on the impeller can significantly improve its hemocompatibility and whether there is a difference between Cit‐IONP‐loaded and non‐loaded ECs in this respect. To this end, the impellers were seeded with Cit‐IONP‐loaded or non‐loaded ECs on the one hand and on the other hand incubated with pooled normal blood plasma (Precision BioLogic) for 6 h to create the clinically relevant control. For the subsequent assay, platelet concentrates from the blood bank of the Hannover Medical School were obtained in accordance with ethical regulations in collection tubes (Sarstedt) containing 1.6 mg EDTA mL^−1^ and spiked with 15 ng mL^−1^ iloprost (Bayer). After a 1:10 dilution with HEP buffer containing another 15 ng mL^−1^ iloprost (Bayer) and a 10 min incubation at room temperature (RT), the suspension was centrifuged (800 x g, 10 min). The platelets were then carefully resuspended in Tyrode buffer and fixed by adding 3 volumes of 4% paraformaldehyde (PFA)/PBS. After 5 min incubation and centrifugation, platelets were resuspended in 1:20 Sudan Black B in 70% ethanol/PBS and stained for 1 h at RT. After centrifugation again, the pellet was washed several times with PBS. The stained platelets were then added to the respective samples at a final concentration of 1 × 10^8^ platelets mL^−1^ and incubated for 1 h at 37 °C on a tilt shaker. Samples were then drawn through a large volume of DPBS and transferred to dishes containing 1 mL DMSO. After 30 min, Sudan Black B of platelets dissolved in DMSO was placed on a 96‐well plate and the optical density was measured at 595 nm using the Synergy‐2 plate reader (BioTek). A serial dilution of known platelet counts in DMSO and empty DMSO applied in parallel to the plate served as a reference to determine the number of platelets adhering per sample. This experiment was conducted in four independent replicates (n = 4).

#### 2.5.3 Step‐by‐Step Approach to Clinical Conditions – Dynamic Incubation Of Statically Pre‐Seeded Impellers

In order to approach an implementation‐relevant application, a successive analysis was chosen in which the influence of dynamic conditions on the endothelialized impeller was quantified via various customized flow reactors.

##### 2.5.3.1 Flow Reactor 1

The first flow reactor (FR1) consisted of an autoclavable 100 mL sample beaker (Simport) modified by adding a central axis embedded in a silicone bottom plate (Remasil, Dentaurum). A central hole was cut in a syringe tap (B.Braun), which was mounted on this axis as a platform for the rotation of the impeller (Figure 5a), allowing for the first dynamic proof of concept.

##### 2.5.3.2 Flow Reactor 2

The second flow reactor (FR2), devised to recreate the flow‐relevant compartment of the original HVAD hosing surrounding the impeller, was designed via Computer‐Aided Design (CAD) (FreeCad© software, version 0.19) and 3D‐printed using the Objet350 Connex3 printer and MED610 biocompatible resin (Stratasys), with SUP706B resin (Stratasys) as the support material, which was removed after printing. Like the original, the printed housing consisted of a top and a bottom part (Figure 5d), which were cleaned after the printing process in a water jet booth, sonicated in isopropanol for 10 min, and cleaned by 70% ethanol and UV treatment (>15 min). For the respective cell culture experiments, the impeller was centered in the housing, then the upper and lower parts were screwed together and placed in a 500 mL glass vessel (Schott) filled with EGM‐2 in order to be able to additionally imitate the gravitational forces relevant in the HVAD housing.

For each experiment, the impellers were seeded with either Cit‐IONP ECs or non‐loaded ECs and cultured for 24 h under static conditions for subsequent use in FR1 and FR2, respectively. The respective flow reactors were then incubated on magnetic stirrers at 1000 rpm (Heidolph MR) for 24 h in a humid atmosphere at 37 °C and 5% CO_2_, with the rotation speed checked by stroboscope. The impellers used for static controls were endothelialized according to the same protocol and placed in the respective flow reactors, but incubated without rotation. In the following analyses, firstly, the viability of the endothelial cells adhering to the impellers was qualitatively assessed using calcein staining (2.4.3.). Using the mask tool of the ImageJ software the area on each impeller blade showing a viable confluent monolayer was measured and the percentage of coverage was calculated in percent. Secondly, comparative quantification of cell number in relation to the different magnetic impeller domains was performed by ROI analysis (see 2.5.1.) as well as PCR analysis (see 2.4.4.) of exemplary primers for shear stress (KLF), coagulation (thrombomodulin), and focal adhesion (vinculin) in comparison between static controls versus dynamic samples of FR1 or FR2 as well as between non‐loaded and Cit‐IONP‐loaded ECs.

##### 2.5.3.3 Original HVAD

In these analyses, a potential translational approach was mimicked by inserting an impeller with an endothelial flow‐conditioned monolayer into the original housing so that the biologized HVAD could be implanted sequentially into the patient. For this purpose, the impeller was positioned in FR1 after 24 h of static endothelialization for preconditioning the ECs at 1000 rpm for 24 h. The impeller was then transferred into sterile DPBS and subsequently installed in the HVAD housing. The unique feature of this translational approach was that care had to be taken during the subsequent assembly of the casing to avoid collisions between the endothelialized surfaces of the impellers and the upper parts of the pump casing due to the repulsive magnetic forces between the impellers and the central posts of the HVAD casings by placing permanent magnets under the lower casing parts when the HVAD was not in operation. The HVAD was then connected to the sterile EGM‐2 filled and vented mock circuit consisting of a glass reservoir with appropriate silicone tubing for extracorporeal circulation (Raumedic) and incubated in a humid atmosphere at 37 °C and 5% CO_2_. With the HVAD controller connected, the minimum pump speed of 1800 rpm was initially applied for 60 min in order to be able to exclude time‐associated effects such as cell division or cell migration during the subsequent evaluation of the calcein images for viability and endothelialization effectiveness. Cell density and percentage EML surface coverage were determined as described in Sections 2.5.1. and 2.5.3.2.

#### 2.5.4 Dynamic Impeller Seeding for the Original HVAD

Since the collision between the endothelialized impeller and the upper parts of the pump housing can lead to potential mechanical cell loss, dynamic impeller endothelialization was tested here to be able to avoid collisions in translational applications. Rotation speed for dynamic endothelialization was set to 400 rpm which resulted in predicted shear stress comparable to the physiological conditions of ECs (see section 2.6.). For this purpose, non‐seeded impellers were mounted in FR1 containing EGM‐2 and set under rotation at 400 rpm. Subsequently, either 2×10^6^ Cit‐IONP‐loaded ECs or non‐loaded ECs were added to the suspension. Samples were analyzed after 4 and 48 h by calcein staining for endothelial viability. The colonization efficiency of adherent ECs was quantified by detecting the cell density per cm^2^ by counting ECs manually in images acquired by fluorescence microscopy of an ROI in the central area of the bearing region (see Figure 7) using the ImageJ software.

### 2.6 Shear Stress Calculations Using Computational Fluid Dynamics

Using STAR‐CCM+ (Siemens PLM Software, version 17.04.007), computational fluid dynamics (CAD) was performed to calculate the putative shear stress generated across the impeller surface during rotation in FR1, FR2 and the original HVAD casing at different speeds. The computational domain was divided into a rotating (impeller) and a static (FR1, FR2 or casing) part. Both parts were discretized with a polyhedral mesh with a base size of 0.5 mm, refining thin parts and boundary layers with a higher resolution. An adaptive mesh model was used for the free surface (FR1, FR2 only). Mesh independence was achieved by refining the mesh until the change in the target value of the wall shear stress was less than 1%. A total of 4.1 million cells formed the computational mesh. The Reynold‐averaged Navier‐Stokes equations were solved on this mesh using a k‐epsilon turbulence model. A segregated flow model was chosen for the transient simulation. An Eulerian multiphase model was used to model the interaction of the two phases (cell suspension and air) in FR1 and FR2. Standard material properties for air were chosen. The viscosity of the cell suspension was set as µ_EGM‐2_ = 0.78 × 10^−3^ Pa × s and the density as ρ_EGM‐2_ = 1003 kg m^−3^. The temperature was set to 37° C. The initial conditions (e.g., filling height of the beaker, non‐rotating rotor) were selected according to the respective experiment.

### 2.7 Statistical Methods

Experiments were conducted with at least three independent replicates (n = 3) and data were expressed as mean ± standard deviation unless otherwise stated. Prism version 9 (Graphpad software) was used for statistical analysis. When comparing two groups, unpaired Student's t‐tests were performed. For three or more groups, one‐way ANOVA tests were used to detect statistical differences. Following this, multiple comparisons were made using either Tukey's post hoc method, when all groups were compared, or Bonferroni's method, when only planned comparisons were made. Statistical significance was indicated with * for *p* < 0.05, ** for *p* < 0.01 and *** for *p* < 0.001.

## 3 Results

### 3.1 Identification of the Optimal Endothelial IONP‐Loading Protocol

**Figure 1:**
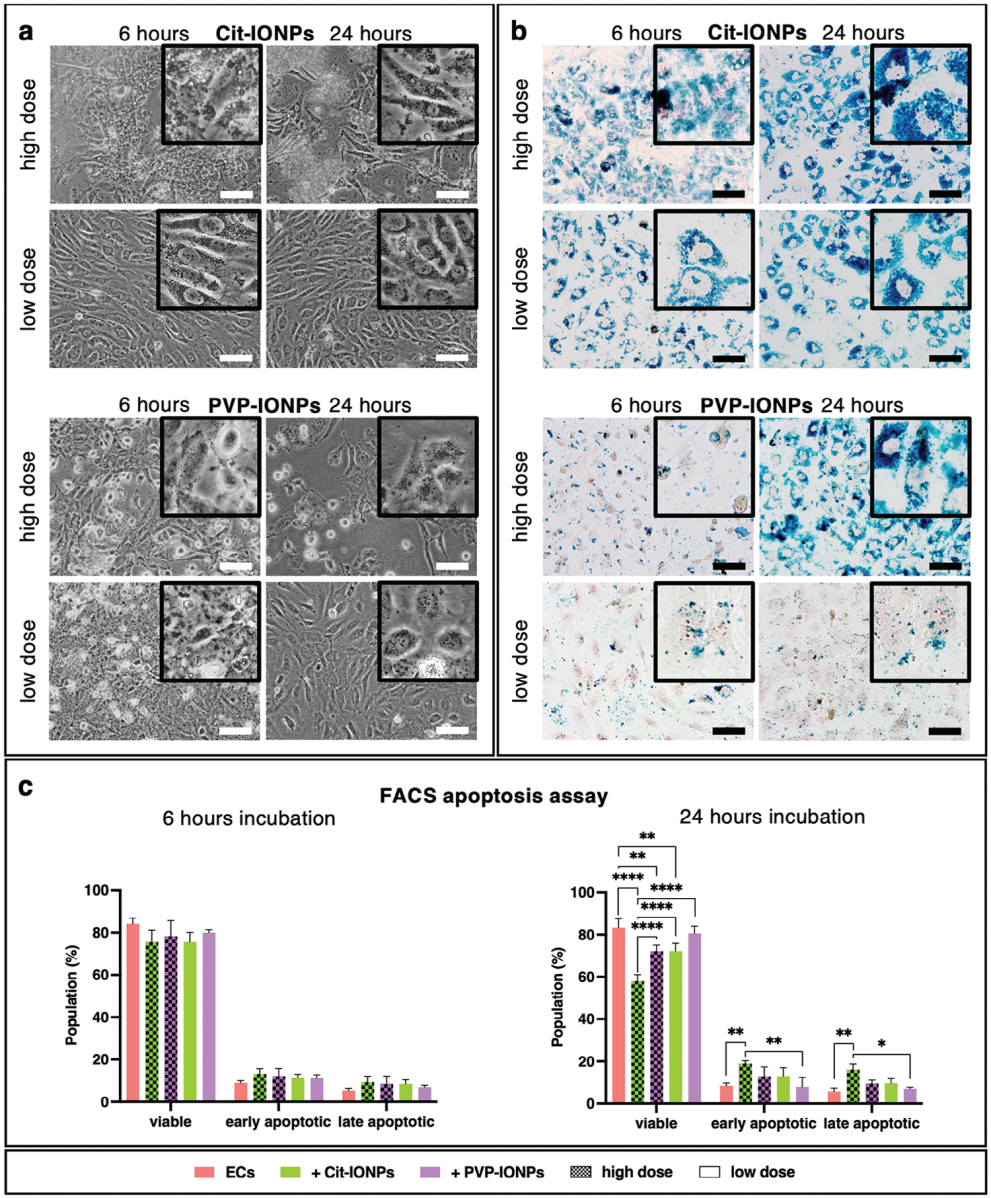
Identification of the optimal endothelial IONP loading protocol. a) phase‐contrast images of EC populations loaded with Cit‐IONPs or PVP‐IONPs at different doses and incubation times; b) Prussian Blue staining of loaded EC populations for the detection of intracellular iron deposits (blue), cell nuclei were counterstained with FastRed (red); scalebars in (a) and (b): 100 µm, insets: 100 × 100 µm; c) Flow cytometric analysis for the detection of apoptotic ECs via annexin V and propidium iodide‐staining. Data are shown as mean with standard deviation. Statistical comparison was carried out using one‐way ANOVA with Bonferroni's multiple planned comparison test (n = 3). Significant differences are indicated by * for *p* < 0.05, ** for *p* < 0.01 and **** for *p* < 0.0001.

**Figure 2:**
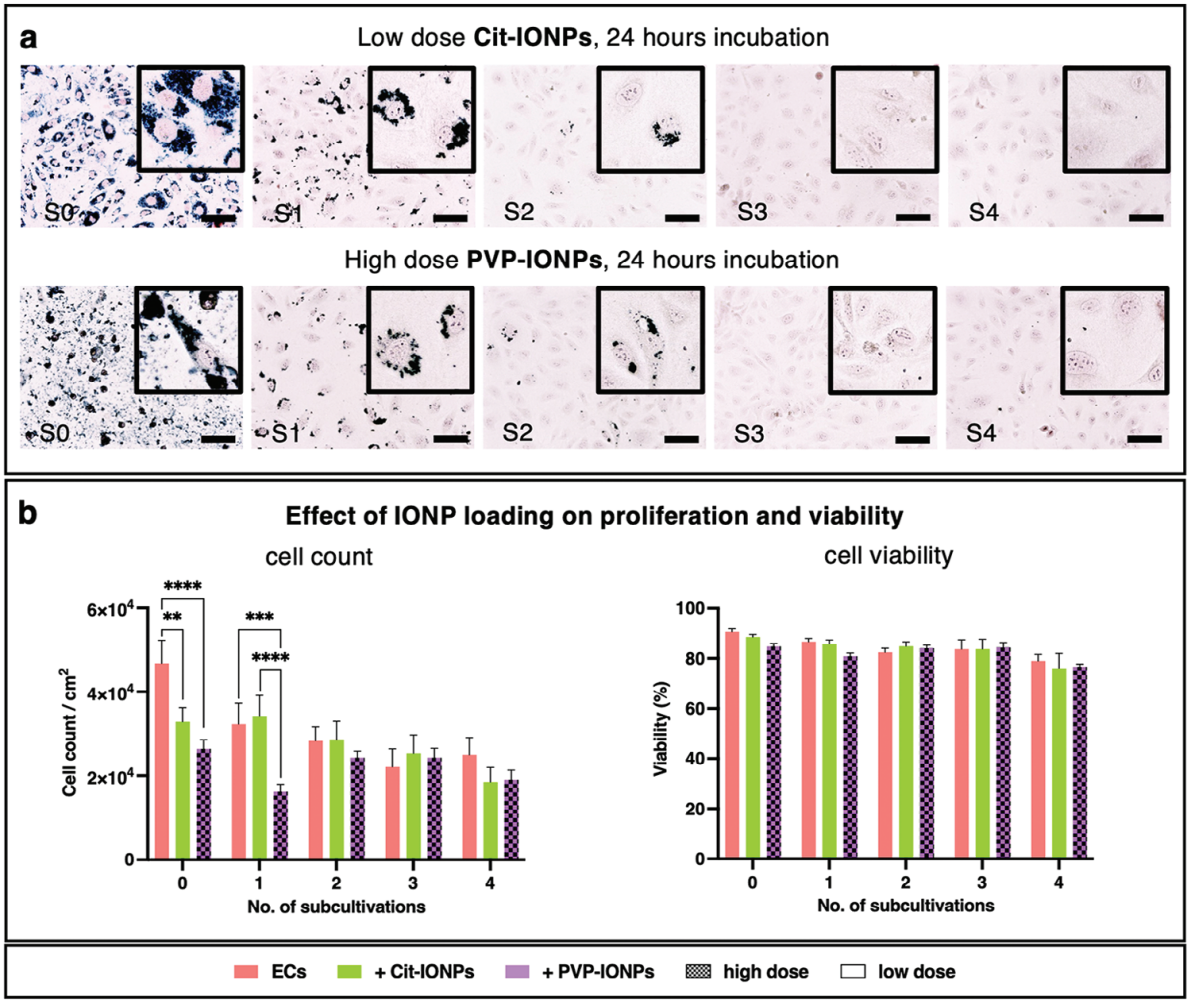
Assessment of residing IONPs during sequential subcultivation and the effect on proliferation and viability. (a) Prussian Blue staining of EC populations upon loading with Cit‐IONP (top row, low dose for 24 h) and PVP‐IONP (bottom row, high dose for 24 h) directly after loading (S0) and at four sequential passages (S1–S4), scalebar: 100 µm, insets: 100 × 100 µm; (b) left: numbers of ECs gained via automated cell counting (CASY) from four consecutive passages after loading with Cit‐IONPs (low dose, 24 h) or PVP‐IONP (high dose, 24 h). right: fraction of viable ECs counted (CASY) at four consecutive passages after IONP loading. Data are shown as mean with standard deviation. Statistical comparison was carried out using one‐way ANOVA with Bonferroni's multiple‐planned comparison test (n = 3). Significant differences are indicated by ** for *p* < 0.01, *** for *p* < 0.001 and **** for *p* < 0.0001.

Data on qualitative endothelial IONP internalization and its influence on cell morphology, apoptosis induction, and proliferation behavior as well as the stability of internalized IONPs under long‐term cultivation were analyzed as part of the determination of the optimal IONP loading protocol. Phase contrast microscopy (**Figure** **1**a) revealed defects in the EML, which depended mainly on the IONP type used (Cit‐IONP vs PVP‐IONP), but also on the applied dose and incubation time, which was particularly pronounced in all ECs internalized with PVP‐IONPs. On the one hand, the endothelial, spindle‐shaped morphology with the formation of the typical cobblestone arrangement was absent here, and on the other hand, a high number of detached ECs was observed. None of these changes were visible in ECs with Cit‐IONPs. In contrast, Prussian blue staining showed that when PVP‐IONPs were used, only the high‐dose, 24‐h incubation resulted in detectable internalization. In comparison, incubation with Cit‐IONPs resulted in distinct intracellular signals, with color intensity increasing as a function of incubation time and Cit‐IONP dosage, reaching its maximum after 24 h of incubation with the high dosage (Figure 1b). Regarding the possible influence of the respective IONP internalization on endothelial apoptosis induction, no significant difference was found for either Cit‐IONP or PVP‐IONP compared to the untreated control group for the 6‐h incubation, also independent of the applied dose (Figure 1c). However, this was different in the 24 h incubation. Here, the percentage of viable ECs exposed to high‐dose Cit‐IONPs was significantly lower (57.94% ± 3.1%) than that of all other populations (all *p* < 0.0001), which was confirmed by an increased percentage of early and late apoptotic ECs. Notably, the percentage of viable ECs was significantly, but only moderately lower after 24‐h exposure to low‐dose Cit‐IONP (72.13% ± 3.75%, *p* = 0.0030) and high‐dose PVP‐IONP (72.13% ± 2.89%, *p* = 0.0029) compared to the untreated control (83.26% ± 4.23%). Based on the morphological data, the efficacy of IONP internalization and the apoptosis assay, only the low‐dose Cit‐IONP and the high‐dose PVP‐IONP, both with 24‐h incubation, were used for the following analyses. Thus, over the long‐term cultivation for 4 cell passages, both the effect of IONP internalization on the proliferation capacity and on the viability of the cells were investigated in comparison to non‐loaded ECs, as well as the possible permanence of this internalization within the framework of the corresponding cell divisions (**Figure** **2**a,b). Here, the decrease in internalized IONPs according to the Prussian blue staining was evident with increasing cell passage, with no qualitative difference between the Cit‐IONPs and the PVP‐IONPs. Both populations were negative for internalized IONPs after the third passage, i.e., on day 9. In terms of proliferation capacity, Cit‐IONP‐ECs showed a trend towards higher cell number immediately after loading (subcultivation 0) compared to PVP‐IONP‐ECs, which was found to be significant after the first subcultivation (3.41 × 10^4^ ± 5 × 10^3^ ECs cm^−2^ vs 16.19 × 10^4^ ± 1.77 × 10^3^ ECs cm^−2^, *p* < 0.0001), indicating better proliferation, while in parallel they also showed higher viability (85.77% ± 1.53% vs 80.83% ± 1.27%, *p* = 0.3268). In the subsequent subcultures, cell counts evened out, so that there were no significant differences among the IONP‐loaded cells, but also not compared to the control cells. It is of note that the cell viability obtained with FACS (Figure 1c) and automated cell counting (Figure 2b) slightly deviated from each other, as no cross‐calibration of the devices was performed.

### 3.2 Cit‐IONP Loading Enables Magnetically Conditioned Cell Guidance While Preserving a Stable Physiological Phenotype

**Figure 3:**
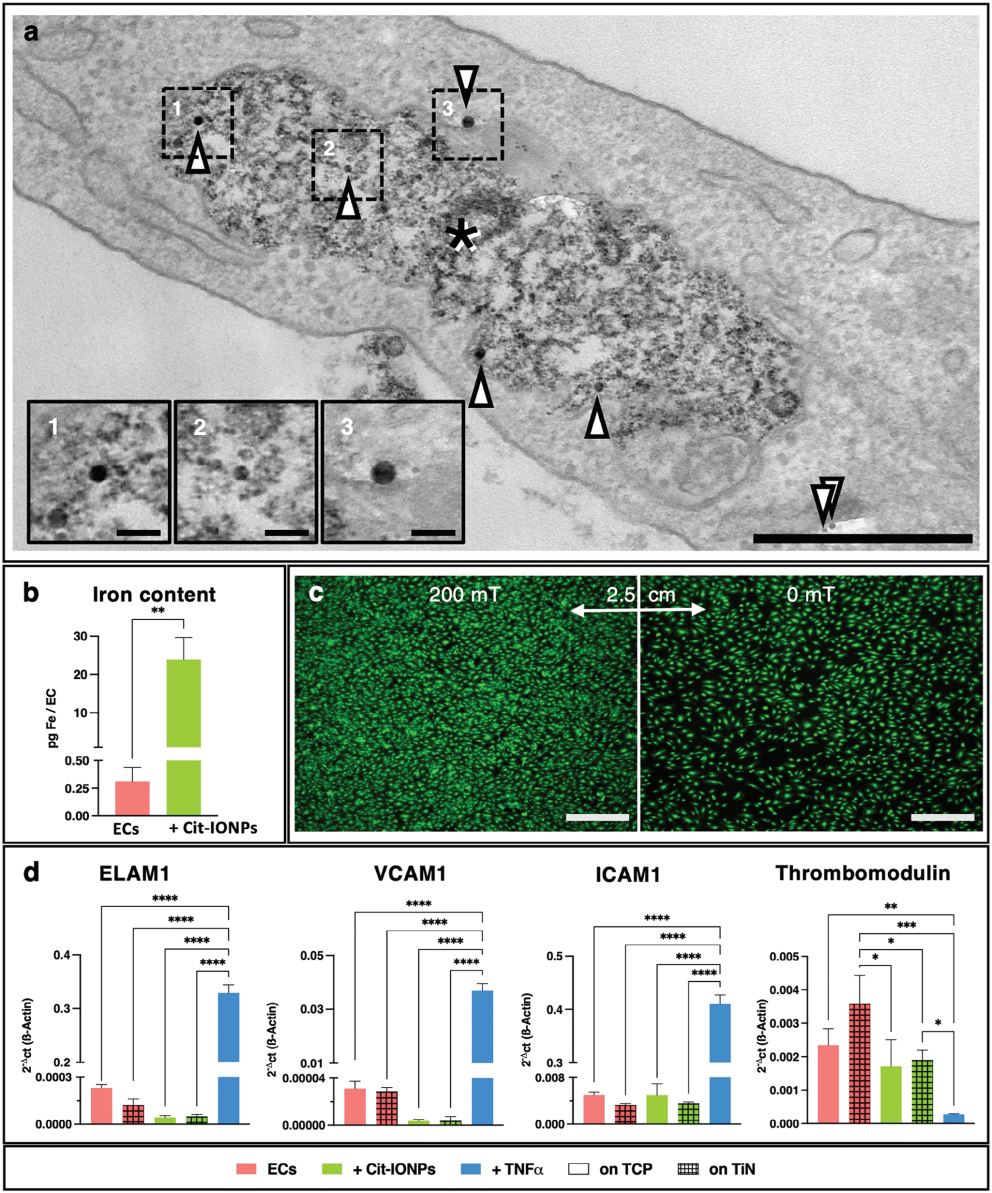
Cit‐IONP loading enables magnetically conditioned cell alignment under stable physiological phenotype. a) TEM image of intracellular IONPs: upward arrowheads indicate IONPs inside autolysosome (asterisk), downward arrowheads indicate IONPs in cytoplasm, Scalebar: 500 nm, insets: larger magnification of single IONPs, (1 and 2) located in the autolysosome, or (3) in the cytoplasm, scalebar: 50 nm; b) Quantification of average iron content per Cit‐ION‐loaded EC via ICP‐OES; unpaired t‐test was used for statistical comparison, with ** for *p* < 0.01 c) Fluorescence microscopy images of calcein‐stained viable ECs (green) on a magnetized TiN coated plate, accumulating at higher density in areas with high magnetic flux (left) compared to low magnetic flux (right). Scalebar: 750 µm. d) qRT PCR analysis for expression changes in pro‐inflammatory and anti‐ thrombogenic genes of ECs seeded on TCP and TiN. Statistical comparison was carried out using one‐way ANOVA with Tukey's multiple comparison test (n = 3). Significant differences are indicated by * for *p* < 0.05, ** for *p* < 0.01, *** for *p* < 0.001 and **** for *p* < 0.0001.

Based on the previous results, subsequent analyses were performed using only the optimal IONP loading protocol, which involved low‐dose Cit‐IONP incubation for 24 h. TEM images verified the uptake and actual intracellular distribution of Cit‐IONP, with the majority of IONPs being smaller than or equal to 25 nm and located in autolysosomal vesicles. Only a small fraction of IONPs were larger than 25 nm or detected in the cytoplasm (**Figure** **3**a). Quantification of intracellular iron content after performing the optimal Cit‐IONP loading protocol showed a mean value of 23.87 ± 5.82 pg iron/cell, which was significantly higher than that of the native control ECs at 0.31 ± 0.13 pg iron/cell (*p* = 0.0022) (Figure 3b). The amount of iron taken up at the low dose protocol for 24 h did not result in mitochondrial dysfunction (see Figure S1a, Supporting Information), as no statistical difference in the number of ECs with intact TMRM‐positive mitochondria was found between non‐loaded and low dose IONP‐loaded ECs (non‐loaded ECs: 91.20 ±0.17% TMRM positive ECs vs low Cit‐IONP ECs: 91.62 ±1.37%, *p* = 0.91). Also, no morphological aberrations, which could have indicated ferroptosis, were found in the mitochondria of loaded ECs (see Figure S1b, Supporting Information).

Mimicking the magnetized impeller surface provided the first evidence for the magnetic attraction of the ECs loaded with Cit‐IONP in this context (Figure 3c). Qualitative evaluation of the fluorescence microscopy images showed that the cell density of viable ECs was higher in the 200 mT range than in the non‐magnetized 0 mT measurement range. Analysis of endothelial gene expression confirmed the stable physiological, non‐inflammatory, and antithrombogenic phenotype which was not significantly affected by Cit‐IONP loading or cultivation on magnetized TiN (Figure 3d). Thus, both non‐loaded and Cit‐IONP‐loaded ECs on TCP and magnetized TiN, respectively, showed the physiological low expression profile with respect to the pro‐inflammatory markers endothelial leukocyte adhesion molecule 1 (ELAM1), vascular cell adhesion molecule 1 (VCAM1) and intercellular adhesion molecule 1 (ICAM1) which were significantly lower in expression than in the TNFα‐induced pro‐inflammatory control ECs (*p*<0.0001, see Table S2, Supporting Information). In the expression analysis of the anticoagulant thrombomodulin (TM), no significant differences were found within the non‐loaded (*p* = 0.13) or Cit‐IONP‐loaded ECs (*p* = 0.99). However, these existed when comparing the non‐loaded ECs on the magnetized TiN and the Cit‐IONP‐loaded ECs, both on TCP (*p* = 0.016) and on the magnetized TiN (*p* = 0.031). With the exception of the Cit‐IONP‐loaded ECs on the TCP (*p* = 0.071), all ECs had significantly higher TM levels compared to the pro‐thrombogenic, TNFα‐stimulated control group.

### 3.3 Magnetic Interaction Between Impeller and Cit‐Ionp Loaded ECs Significantly Increased the Cell Density of the Physiological Confluent Monolayer, Resulting in Significantly Reduced Platelet Adhesion

**Figure 4:**
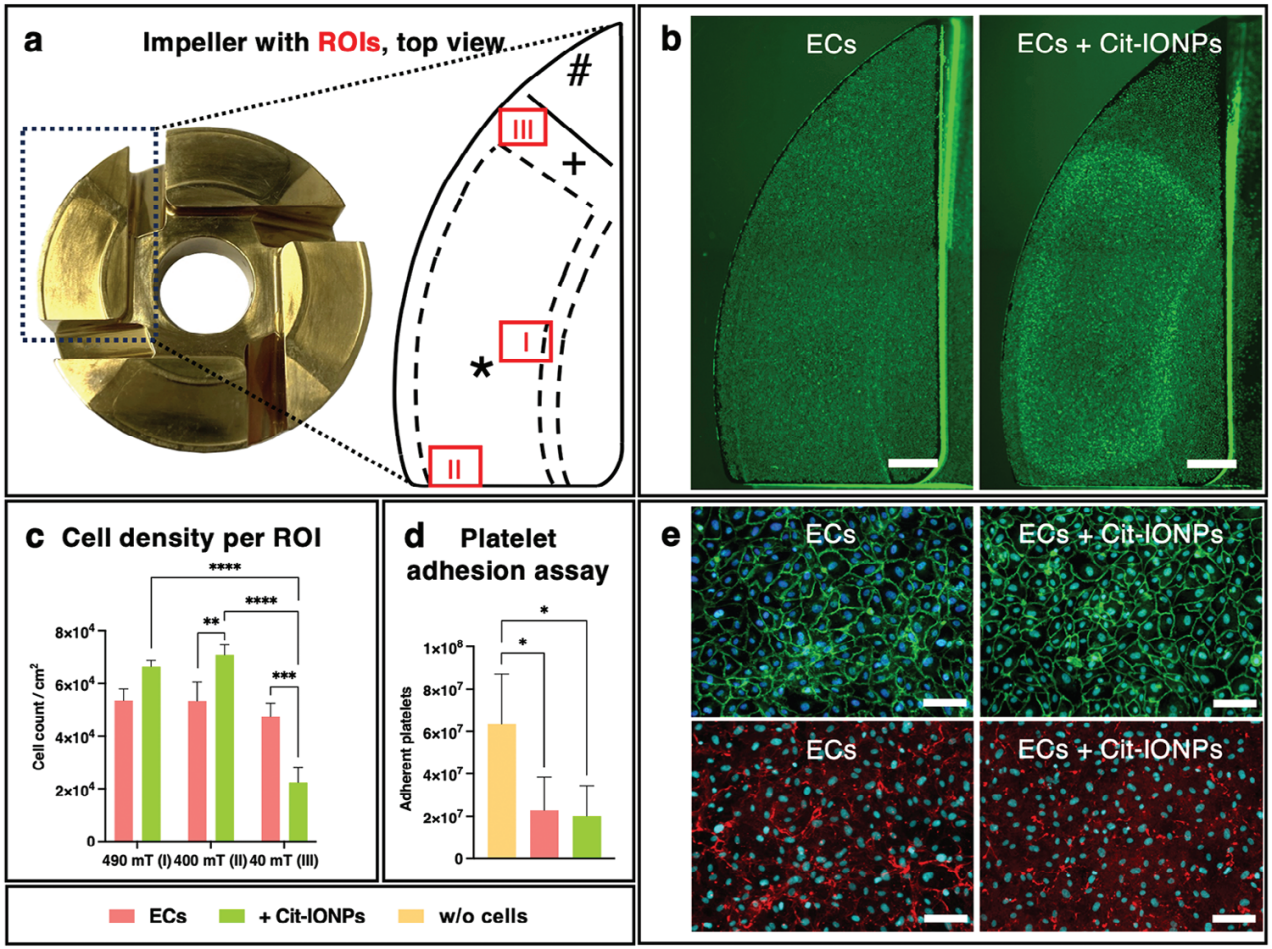
Magnetic interaction between impeller and Cit‐IONP loaded ECs significantly increased the cell density of the physiological confluent monolayer, resulting in significantly reduced platelet adhesion. a) Top view of the HeartWare VAD impeller and a schematic indication of selected ROIs with different magnetic field strengths. ROI I: 490 mT, close to the bottom ridge of the bearing region (*), ROI II: 400 mT, close to the axial boundary of the bearing surface, ROI III: 40 mT in the bridge (+) region between the bearing surface and the pressure relief (#); b) fluorescence images of calcein stained viable monolayers (green) on impellers after seeding with non‐loaded ECs (left) or Cit‐IONP‐loaded ECs (right); scalebar: 1 mm; c) quantification of cell density of non‐loaded and Cit‐IONP‐loaded ECs in selected ROIs; Statistical comparison was carried out using one‐way ANOVA with Bonferroni's multiple planned comparison test (n = 3). Statistical significance was indicated with ** for *p* < 0.01, *** for *p* < 0.001 and **** for *p* < 0.0001. d) platelet adhesion assay with non‐loaded and Cit‐IONP‐loaded ECs; one‐way ANOVA with Tukey's multiple comparison test (n = 4), with * indicating significant difference with *p* < 0.05; e) immunofluorescence microscopy of monolayers established from non‐loaded ECs (left column) and Cit‐IONP‐loaded ECs (right column) for the detection of VE‐Cadherin (green) and collagen type IV (red), nuclei were counterstained with Hoechst 33342 (blue), scalebar: 100 µm.

Each of the impeller's four blades contains a permanent magnet. On the impeller blade surface, three distinct areas (ROI) were defined based on magnetic flux density measurements and surface geometry (**Figure** **4**a): ROI I (490 mT) is located in the exit area and ROI II (400 mT) in the entry area of the bearing surface, while ROI III (40 mT) is located in the transition from the plane bridge to the pressure relief area.^[^ ^41^ ^]^ The overview images of both the non‐loaded and the Cit‐IONP‐loaded ECs seeded onto the impellers show qualitatively completely endothelialized impeller blade surfaces (Figure 4b). However, applying the three ROIs with the different magnetic strengths to the overview images, one sees a condensed calcein signal in the highly magnetic ROIs I and II and an attenuated signal for ROI III with the Cit‐IONP ECs while a uniform calcein signal is seen for the non‐loaded ECs. Immunofluorescence staining of the two EMLs on the impeller blades against VE‐cadherin (green), an endothelial‐specific cell‐cell adhesion protein, and collagen IV (red), an integral component of the basal lamina, supports this (Figure 4e). On the one hand, both monolayers showed comparable overall signal levels of VE‐cadherin at the individual cell junctions, correspondingly confirming confluence and characteristic cobblestone morphology. On the other hand, collagen IV was comparably detected under the non‐loaded and Cit‐IONP‐loaded EC monolayers. However, besides the evidence of comparable integrity and quality of the monolayers, there was an obvious condensation of the corresponding signals in Cit‐IONP‐loaded monolayers, analogous to the observations of calcein staining. These qualitative impressions could be confirmed by quantifying the cell density per ROI (Figure 4c). In general, the cell numbers of the non‐loaded cells did not differ significantly between the three ROIs (mean cell number = 5.14 × 10^4^ ± 0.33 × 10^4^ ECs cm^−2^), while the density of Cit‐IONP ECs was significantly higher in the highly magnetic regions ROI I (6.62 × 10^4^ ± 2.59 × 10^3^ ECs cm^−2^) and II (7.08 × 10^4^ ± 3.78 × 10^3^ ECs cm^−2^) than in the least magnetic ROI III (2.24 × 10^4^ ± 5.8 × 10^3^ ECs cm^−2^, *p* < 0.0001). When comparing the two cell populations, it was found that cell density was significantly higher for the non‐loaded ECs in ROI III (*p* = 0.0005), but lower in the highly‐magnetic ROI I and II, the latter comparison yielding a significant advantage for the Cit‐IONP‐ECs (*p* = 0.082 and *p* = 0.0098, respectively).

The basic hypothesis that endothelialization of the impeller leads to significantly improved hemocompatibility was confirmed with the following platelet adhesion test (Figure 4d), in which the respective number of platelets adhering to the different impellers was determined and compared. No significant differences were found between the endothelialized impellers, regardless of whether they were populated with non‐loaded or Cit‐IONPs loaded ECs (2.28 × 10^7^ ± 1.55 × 10^7^ thrombocytes and 2.02 × 10^7^ ± 1.42 × 10^7^ thrombocytes, respectively, *p* = 0.9772). A distinct and significant difference between the endothelialized impellers and the clinical control, the impeller incubated with blood plasma, which had a significantly higher number of adherent platelets (6.34 × 10^7^ ± 2.36 × 10^7^ thrombocytes, *p* = 0.01289 and *p* = 0.0210, respectively) was measured.

### 3.4 Dynamic Flow Conditions Confirm Significantly Increased Adherence of Cit‐Ionp‐ Loaded ECs By Magnetic Interaction, Which Continue to Exhibit Physiological Behavior

**Figure 5:**
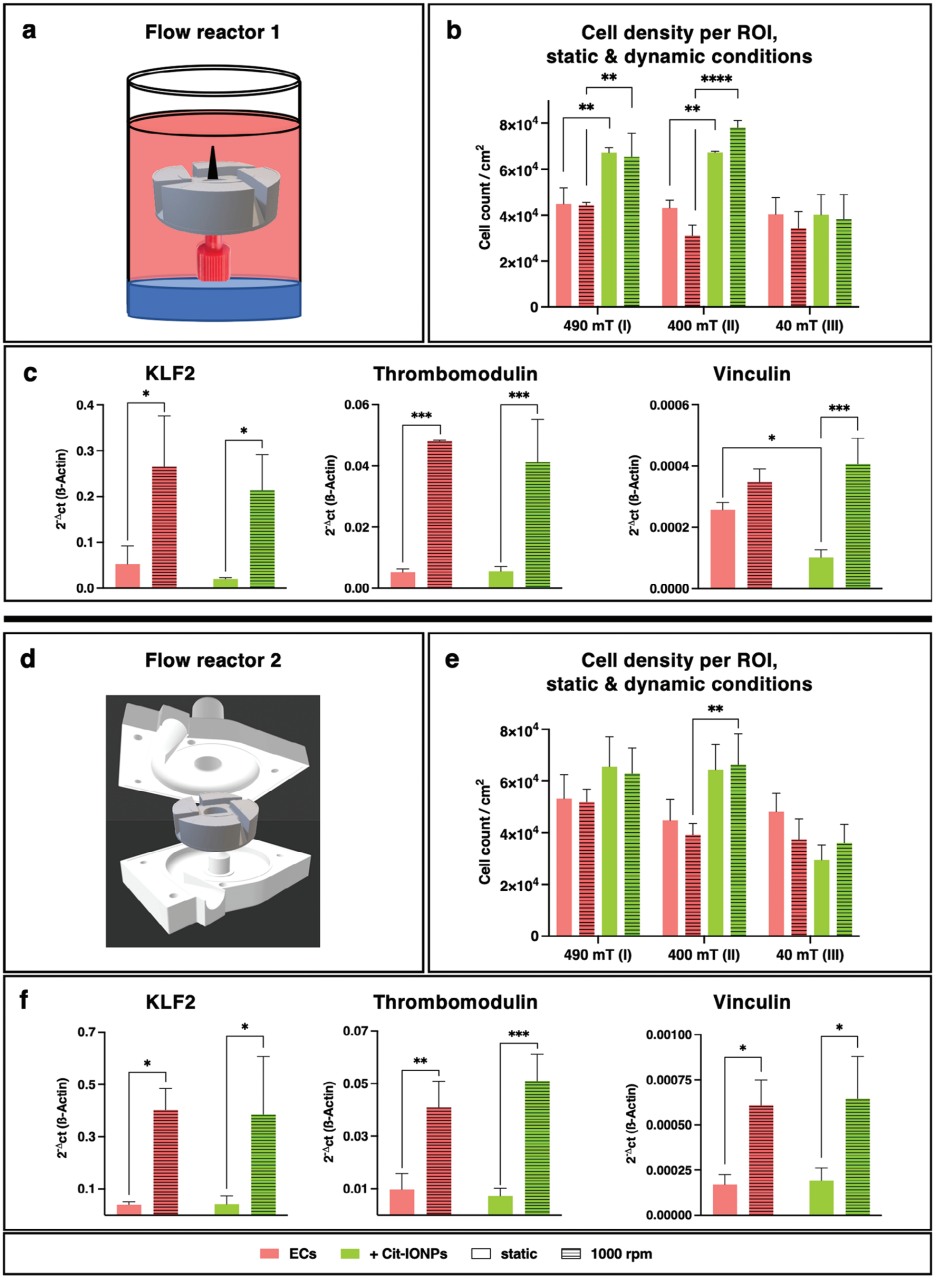
Dynamic flow conditions confirm significantly increased adherence of Cit‐IONP‐loaded ECs by magnetic interaction, which continue to exhibit physiological behavior. a,d) Schematic view of flow reactor 1 (a: medium‐filled sample container) and flow reactor 2 (d: reverse engineered 3D printed HVAD housing), where impellers rotated at 1000 rpm; b,e) Quantification of the cell density in selected ROIs under static and dynamic conditions; c,f) Expression analysis of marker genes indicating physiological response to shear stress (KLF2, Thrombomodulin, Vinculin). Statistical significance was assessed using one‐way ANOVA and Bonferroni's multiple‐planned comparison test (n = 3). Significant differences are indicated by * for *p* < 0.05, ** for *p* < 0.01, *** for *p* < 0.001 and **** for *p* < 0.0001.

The first fluid dynamic analyses were carried out with the two self‐constructed bioreactors (**Figure** **5**a,d) in which the endothelialized impellers rotated at 1000 rpm for 24 h each. The construction of FR1 resulted in a calculated mean shear stress of 10.61 ± 4.74 Pa (max. 106.1 Pa). For FR2, in which the 3D‐printed HVAD housing surrounded the impeller, the values were 124.37 ± 139.29 Pa (max. 2099.14 Pa). In the following, the respective EC densities of the non‐loaded and Cit‐IONP‐loaded ECs within the three different ROIs on the impellers after rotation were quantified and evaluated in comparison to the static controls (Figure 5b,e). When FR1 with the lower shear rate was operated, there were no significant differences within the group of non‐loaded (ROI I: 4.4 ± 0.11 × 10^4^ cm^−2^, ROI II: 3.12 ± 0.45 × 10^4^ cm^−2^, ROI III: 3.44 ± 0.73 × 10^4^ cm^−2^) or Cit‐IONP‐loaded ECs (ROI I: 6.56 ± 1.16 × 10^4^ cm^−2^, ROI II: 6.73 ± 0.06 × 10^4^ cm^−2^, ROI III: 3.8 ± 1.06 × 10^4^ cm^−2^) of the different ROIs compared to the respective static controls (non‐loaded ROI I: 4.45 ± 0.11 × 10^4^ cm^−2^, ROI II: 4.30 ± 0.35 × 10^4^ cm^−2^, ROI III: 4.04 ± 0.72 × 10^4^ cm^−2^; Cit‐IONP‐loaded ROI I: 6.72 ± 0.20 × 10^4^ cm^−2^, ROI II: 6.73 ± 0.06 × 10^4^ cm^−2^, ROI III: 3.44 ± 0.71 × 10^4^ cm^−2^). The same was observed when FR2 with the higher shear rate was used. Here, also no significant differences within the group of non‐loaded (ROI I: 5.20 ± 0.485 × 10^4^ cm^−2^, ROI II: 3.92 ± 0.82 × 10^4^ cm^−2^, ROI III: 3.75 ± 0.78 × 10^4^ cm^−2^) or Cit‐IONP‐loaded ECs (ROI I: 6.30 ±0.98×10^4^ cm^−2^, ROI II: 6.64 ±1.18×10^4^ cm^−2^, ROI III: 3.60 ±0.73×10^4^ cm^−2^) of the different ROIs compared to the respective static controls (non‐loaded ROI I: 5.33 ±0.93×10^4^ cm^−2^, ROI II: 4.48 ±0.82×10^4^ cm^−2^, ROI III: 4.82 ±0.78×10^4^ cm^−2^; Cit‐IONP‐loaded ROI I: 6.56 ±1.16×10^4^ cm^−2^, ROI II: 6.43 ±0.98×10^4^ cm^−2^, ROI III: 2.95 ±0.57×10^4^ cm^−2^) were detected. However, a significant difference was observed in both FR1 and FR2 between the non‐loaded and Cit‐IONP‐loaded ECs. In FR1, this was in ROIs I and II with a significantly higher cell density of the Cit‐IONP‐loaded ECs compared to the non‐loaded ECs, both in the static controls (see also Figure 4d) and in the dynamic samples. Under the dynamic conditions in FR2, the cell densities of the Cit‐IONP‐ECs were significantly higher in ROI II compared to the non‐loaded ECs (*p* = 0.0074). Both flow reactors showed comparable cell densities of the different ECs for the low magnetic area ROI III, under both static and dynamic conditions.

To describe the total retention of ECs over the entire impeller surface, the cell numbers of all ROIs per impeller were calculated and compared between the two different flow reactor setups, which were used to investigate, among other things, the endothelial adherence of non‐loaded and Cit‐IONP‐loaded ECs. In the FR1 setup, the total number of retained cells was significantly higher when IONP‐ECs were applied to the impeller compared to non‐loaded ECs (6.07 × 10^4^ ± 1.92 × 10^4^ IONP‐ECs cm^−2^ vs 3.67 × 10^4^ ± 7.38 × 10^3^ ECs cm^−2^, *p* = 0.0029). This effect was also observed in the FR2 setup, but without statistical significance (5.51 × 10^4^ ± 1.67 × 10^4^ IONP‐ECs cm^−2^ versus 4.29 × 10^4^ ± 8.52 10^3^ ECs cm^−2^, *p* = 0.0684). Overall, the fluorescence microscopy calcein images of the respective impellers still showed viable and confluent EMLs after rotation, especially for the ECs loaded with Cit‐IONP (see Figures S3a and S4a, Supporting Information). In addition, qRT‐PCR analyses of the ECs were performed after each rotation or static control incubation within FR1 and FR2 to quantify possible differences in the regulation of genes associated with shear stress (Figure 5c,f). In both FR setups, expression of shear stress‐dependent Krüppel‐like factor 2 (KLF2) and antithrombogenic thrombomodulin (TM) within the respective groups of non‐loaded and Cit‐IONP‐loaded ECs was significantly higher in the dynamic samples when compared to their respective static controls (see Table S3, Supporting Information) but this was not found when the two cell populations (Cit‐IONP‐ECs vs native ECs) were compared to each other. For focal adhesion‐associated vinculin (VCL) this was also the case, with the exception of non‐loaded ECs in FR1, which did not show significant upregulation when rotated at 1000 rpm. Also, under static conditions, VCL was significantly but negligibly less expressed in the tested Cit‐IONP ECs in FR1 (*p* = 0.0202, see Table S3, Supporting Information). This difference was not observed in FR2, although the non‐loaded and Cit‐IONP‐ECs were exposed to the same conditions.

The levels of shear‐stress‐related gene expression in samples rotated in the two different reactors were also compared within the two cell populations. Although we noted that the levels of expression tended to be higher in samples from FR2 with its higher shear, this difference was only significant for the comparison of VCL expression in non‐loaded ECs from FR1 versus non‐loaded ECs from FR2 (*p* = 0.0362).

### 3.5 Translationally Relevant HVAD Application with Statically Pre‐Endothelialized and Dynamically Preconditioned Impellers Resulted in Significant Cell Loss

**Figure 6:**
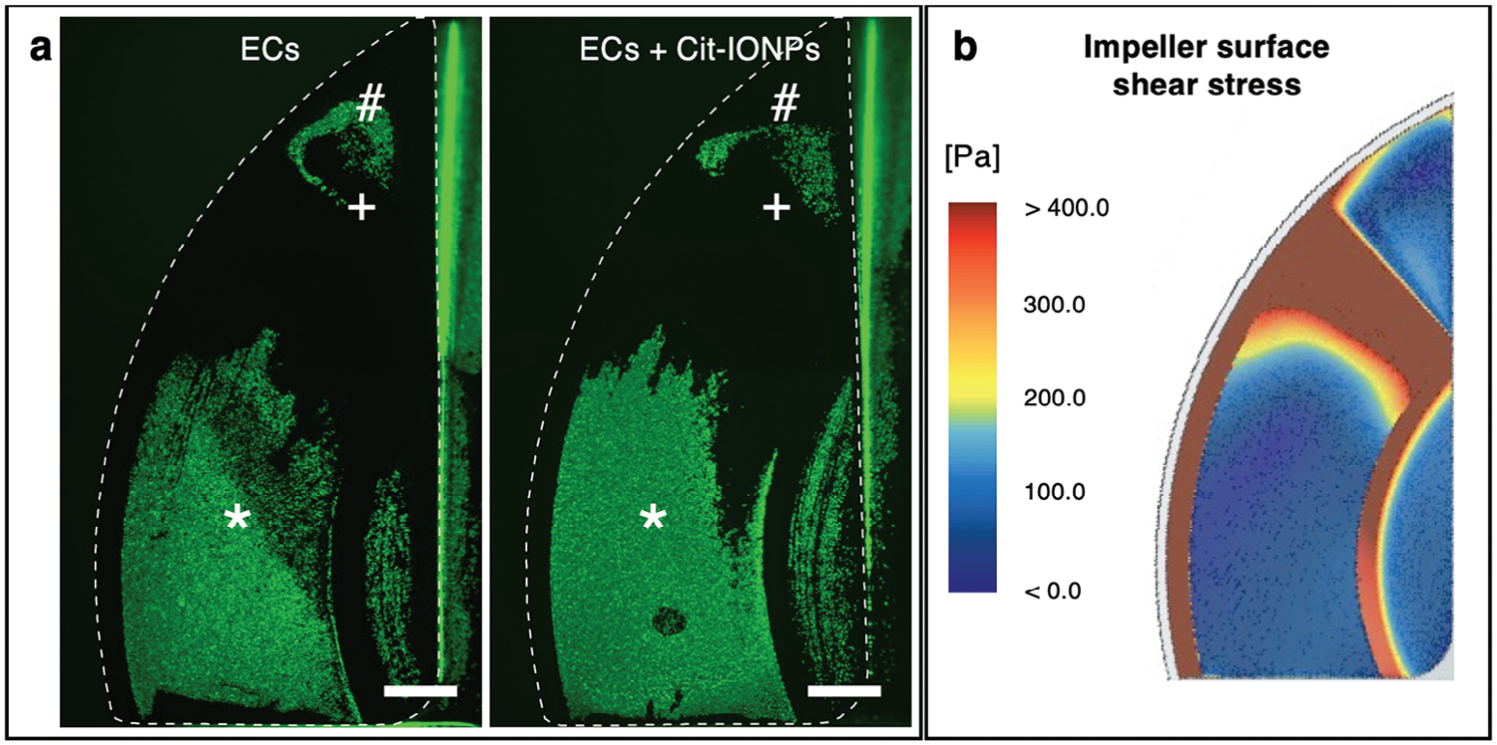
Translationally relevant HVAD application with statically pre‐endothelialized and dynamically preconditioned impellers resulted in significant cell loss. a) Fluorescence microscopy image of calcein‐stained viable ECs (green) without (left) or with Cit‐IONPs (right) after 60 min rotation inside original HVAD. White broken line indicates impeller blade ridge; (*) marks the bearing surface, (+) marks the bridge region, (#) marks the pressure relief; Scalebar: 1 mm; b) CFD‐calculated heat map of shear stress distribution on impeller surfaces inside original HVAD housing at 1800 rpm.

In comparison to the proof‐of‐concept investigations mentioned in 3.4., here impellers were pre‐incubated with non‐loaded or with Cit‐IONP‐loaded ECs and flow‐conditioned to subsequently undergo a 1 h rotation at 1800 rpm in the original HVAD housing. In the context of pre‐flux and post‐flux impeller assembly, the special feature of this translationally relevant approach was that collision between the endothelialized impeller surfaces and the upper parts of the HVAD housing due to the repulsive magnetic forces had to be prevented by additional permanent magnets under the lower housing parts. The mean shear stress calculated for this application across the impeller surface was 225.42 ± 251.70 Pa with few areas, where a maximum of 5451.28 Pa was reached. The qualitative analysis of viable and adherent ECs on the calcein‐stained impellers after flow application showed significant cell loss from the original confluent monolayer for both non‐loaded and IONP‐loaded ECs (**Figure** **6**a). Comparison of the heatmap of the CFD‐calculated shear stress on the impeller surface with the adherence of the respective ECs showed the viable ECs only in the bluish‐colored areas with <180 Pa (Figure 6a), i.e., the majority of the remaining ECs were seen in the entrance region of the bearing surface, in the semilunar‐shaped region adjoining to the central hole and in the pressure relief region (see also Figure 4a). Quantitative evaluation of the impeller areas still endothelialized after flow revealed 30.25% for the non‐loaded ECs and 39.1% for the IONP‐loaded ECs (see Figure 6), which also showed an appreciably higher cell density compared to the non‐loaded ECs in ROIs I and II, the impeller regions with high magnetic field strength (see Figure S5, Supporting Information).

### 3.6 Clinically Compliant Protocol: Formation of a Flow Resistant Monolayer After Seeding Cit‐IONP‐Loaded ECs on Rotating Impeller at Physiological Shear Stress

**Figure 7:**
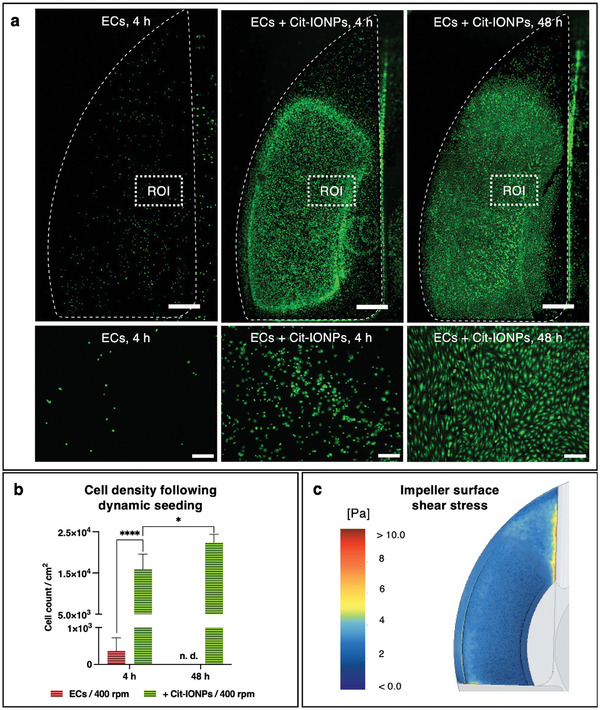
Conceivable clinical protocol: Dynamic impeller seeding under physiological shear rates enabled the formation of a confluent, flow‐resistant monolayer with the Cit‐IONP‐loaded ECs. a) Fluorescence microscopy images of calcein‐stained viable cells on impellers 4 or 48 h after seeding under rotation in FR1 at 400 rpm. Top row: total view on top of one representative impeller blade, scalebar: 1 mm, white broken line indicates impeller blade ridge, bottom row: larger magnification of ROI of above‐shown impeller surface, scalebar: 250 µm. b) Quantification of cell density in the central area of impeller 4 and 48 h after seeding wither with Cit‐IONP loaded or non‐loaded ECs; n.d. = not detectable. c) Heat map view of CFD‐calculated shear stress distribution on impeller at 400 rpm in FR1 and culture medium. Statistical comparison was carried out using one‐way ANOVA with Bonferroni's multiple‐planned comparison test (n = 3). Significant differences are indicated by * for *p* < 0.05 and **** for *p* < 0.0001.

During dynamic endothelialization of the impeller at 400 rpm in FR1, mean shear rates of 3.22 ±1.25 Pa occurred under the experimental conditions, with a maximum of 27.14 Pa, showing a nearly homogeneous distribution of <4 Pa over the whole impeller surface, based on the heatmap of CFD‐calculated shear stress (**Figure** **7**c). Quantitative analysis of the viable and adherent ECs after 4 h of rotation showed clear differences between the non‐loaded and Cit‐IONP‐loaded ECs. Only a small number of non‐loaded ECs were detected on the impeller surface, which also remained spherical and did not form cell‐cell contacts. In comparison, markedly more Cit‐IONP‐loaded ECs were found on the impeller surface, which was already morphologically flattened, showed increasing cell density, especially in the highly magnetized regions (ROI I and II) and started to form cell clusters (Figure 7a). In contrast, in the low‐magnetized region (ROI III), only isolated adherent cells appeared even in the ECs loaded with Cit‐IONP. After 48 h of dynamic incubation, no viable non‐loaded ECs could be detected. In contrast, the viable, confluent monolayer of Cit‐IONP‐loaded ECs was, on the one hand, more homogeneously distributed and, on the other hand, an increasing endothelialization of the previously hardly colonized low‐magnetic region ROI III could be detected. Quantitative enumeration of adherent ECs on the respective impeller surfaces showed significant differences between the non‐loaded and the Cit‐IONP‐loaded ECs after both 4 h (0.37 × 10^3^ ± 0.35 × 10^3^ ECs cm^−2^ versus 1.59 × 10^4^ ± 3.67 × 10^3^ IONP‐ECs cm^−2^, *p* < 0.0001) and 48 h (2.23 × 10^4^ ± 2.01 × 10^3^ IONP‐ECs cm^−2^, *p* = 0.011) of rotation (Figure 7b).

## 4 Discussion

Although endothelialization of blood‐contacting implants is an effective strategy to significantly improve their hemocompatibility,^[^ ^24^, ^27^, ^28^ ^]^ there is only little data on its application in LVAD systems to date.^[^ ^31^ ^]^ However, the transfer of this strategy to the impeller surface is of tremendous clinical importance as this is the area with the highest risk of thrombus formation (see Figure S2, Supporting Information). At the same time, it is also the area with the highest shear rates.^[^ ^32^, ^33^ ^]^ These also define the high requirements for effective endothelialization, where the EML must directly withstand these supraphysiological hemodynamic forces, which cannot be guaranteed with current endothelialization protocols. With the proof of concept described in this study, we were able to effectively address this challenge for the first time by exploiting the ability to induce cells with incorporated IONPs in a magnetic field. Here, the attraction of Cit‐IONP‐loaded ECs to the highly magnetic surface of the impeller resulted in significant endothelialization that met demanding hemodynamic conditions and therefore may be transferred to other magnetic LVAD systems (e.g., HeartMate3) in the future.^[^ ^34^ ^]^ The experiments were performed with primary ECs to demonstrate and ensure comparability with other seeding protocols and thus also their validity and reproducibility.^[^ ^27^, ^35^ ^]^ On the other hand, it is a clinically relevant cell source with which ECs can be generated in sufficient quantities compared to cells of autologous origin and which can be made immunologically invisible by genetic modification to be recognized as autologous cells by the immune system.^[^ ^31^, ^36^ ^]^

### 4.1 Biocompatibility of Nanoparticle Coating for Optimal Cellular Internalization

According to current literature, the efficiency of intracellular uptake of IONPs varies by cell type and depends on the size, coating, concentration, and incubation time of the nanoparticles used.^[^ ^37^, ^38^, ^39^ ^]^ In the first approach, the optimal incubation time of IONPs with an average size of 25 nm, originally characterized as biocompatible, was determined with two different coatings, namely electrostatically stabilized anionic Cit‐IONPs^[^ ^40^, ^41^ ^]^ and sterically stabilized PVP‐IONPs,^[^ ^42^, ^43^ ^]^ as a function of their concentration. Already the use of low‐dose Cit‐IONPs led to a clear Prussian blue signal after 6 h of incubation as evidence of intracellular iron uptake. The intensity of the staining correlated with the incubation time and the concentration of Cit‐IONP. In contrast, a significant iron signal for internalized PVP‐IONP could only be detected after 24 h of high‐dose administration, which reflected the findings of earlier studies exploiting the PVP coating a steric repulsion tool to inhibit nonspecific interactions between nanoparticles and cells, particularly to increase the in vivo circulation time upon intravenous injection, e.g., as MRI contrast agents.^[^ ^44^ ^]^ However, a high dose of PVP‐IONP incubation resulted in a strong change in cell morphology and abundant detached cells in the supernatant and was interpreted as a sign of acute cell death. Removed with the supernatant, necrotic cells could not be included in the FACS analysis so after 24 h of PVP‐IONP exposure, fewer apoptotic cells were detected than in populations exposed to high doses of Cit‐IONP over the same period, which showed significantly reduced cell viability below the 70% threshold defined in the ISO10993‐5 standard for biocompatibility testing,^[^ ^45^ ^]^ so that this approach was considered as not biocompatible. In addition, 66 µg iron mL^−1^ Cit‐IONPs clearly exceeded the maximum amount of iron of approx. 25 – 50 µg mL^−1^ described as tolerable for cells.^[^ ^37^, ^44^, ^46^, ^47^, ^48^ ^]^ Although the group of high‐dose PVP‐IONPs with 48.5 µg iron mL^−1^ was by definition within this biocompatibility range, we interpreted the FACS analysis as falsely high, since significantly less viable ECs were detected compared to the control ECs and the Cit‐IONPs group in the subsequent proliferation and viability tests so that this approach was also considered as causing cytotoxicity. It is of note that the discrepancy in percental viability between the flow cytometric apoptosis assay and the subcultivation experiment may have been caused by using two different devices, which were not cross‐calibrated. The sensitive flow cytometry assay may have detected cells already in a very early stage of apoptosis, where the cells still provided enough membrane resistance to be considered viable in the automated cell counter. However, since 24 h incubation with 6.6 µg mL^−1^ low‐dose Cit‐IONPs led to significant but not cytotoxic internalization of IONPs, this protocol was chosen for all further experiments.

A possible explanation for the reduction in viability of Cit‐IONP‐ECs may be ferroptosis, an iron‐dependent cell death pathway distinct from apoptosis and necrosis, which has been reported to occur in IONP‐treated ECs, depending on surface coating and the extent of cellular uptake.^[^ ^49^, ^50^ ^]^

Indeed, when investigating the mitochondrial membrane potential following Cit‐IONP loading for 24 h it was found that high doses of Cit‐IONPs induced severe depolarization and loss of mitochondria function (see Figure S1a, Supporting Information). In contrast, the low‐dose protocol only had negligible effects on mitochondrial membrane potential and no appreciable impact on mitochondrial morphology (see Figure S1a,b, Supporting Information), which was in accordance with current literature, reporting that ferroptosis was dependent on the type of coating and extent of IONP uptake.^[^ ^50^, ^51^, ^52^ ^]^ While the low‐dose protocol chosen for further experiments remained under the cytotoxicity threshold, efforts to better understand the cell death mechanisms as well as further fine‐tuning of the ideal IONP concentration in future studies might lead to a revised IONP loading process with improved cell viability.

However, the IONP loading did result in a measurable difference in cell growth between the groups that persisted until the third subpopulation, after which the cells were no longer positive for iron staining. On the one hand, the IONPs were distributed to the respective daughter cells during cell proliferation and thus diluted,^[^ ^53^ ^]^ but this is clinically negligible, as cell proliferation within a confluent monolayer is not to be expected due to physiological contact inhibition of the ECs. On the other hand, the IONPs were released into the cell medium during cell division,^[^ ^54^ ^]^ which in a future clinical setting would mean that IONPs would be released into the patient's blood. However, IONPs are unlikely to have a harmful systemic effect, as their safety has already been confirmed in in‐vivo tests^[^ ^54^, ^55^ ^]^ and they are also already approved for other therapeutic clinical applications, e.g., as a contrast‐enhancing agent for magnetic resonance imaging^[^ ^56^ ^]^ or for the treatment of iron deficiency anemia.^[^ ^57^ ^]^ Although the decreasing intracellular IONP concentration with each cell division also reduces the magnetic attraction, this is only required initially anyway, as the ECs synthesize their extracellular matrix on the abluminal^[^ ^58^ ^]^ and blood‐facing side^[^ ^59^ ^]^ de novo, depending on the prevailing flow conditions, to effectively resist the excessive shear forces.

### 4.2 Effective IONP Internalization Ensures Physiological, Magnetically Guided EML

The continued suitability of low‐dose Cit‐IONPs was demonstrated by TEM, which showed a size of the intracellular nanoparticles well below the superparamagnetic limit of 30 nm.^[^ ^60^ ^]^ This was considered important for the putative clinical application, as larger nanoparticles would own magnetic remanence even after exiting the magnetic field, potentially leading to the downstream aggregation of cells released into the bloodstream, which could cause complications in the patient (e.g., embolic occlusions).^[^ ^55^, ^60^, ^61^ ^]^ ICP‐OES revealed an average iron concentration of 23.87 pg iron per EC, falling within the non‐toxic range of 16 – 38 pg iron per EC,^[^ ^47^, ^62^, ^63^ ^]^ which was sufficient to significantly direct the ECs into the higher magnetic flux density region on a TiN‐coated surface, as reported in previous studies.^[^ ^64^, ^65^ ^]^ Neither magnetic attraction above 200 mT nor the TiN surface, both features of the original impeller surface, affected the viability or physiological non‐inflammatory and non‐thrombogenic gene expression profile of ECs, which is consistent with current literature.^[^ ^66^, ^67^, ^68^, ^69^ ^]^ In contrast, to the results of *Ge* et al., who also used IONPs but whose coating consisted of di‐mercaptosuccinic acid, no significant upregulation of the proinflammatory genes ICAM‐1, VCAM‐1, and ELAM‐1 was observed in cells using our internalization protocol.^[^ ^70^ ^]^ As for antithrombogenic thrombomodulin, although a slight but significant upregulation of non‐loaded ECs was observed on TiN, TM expression of Cit‐IONP‐ECs was significantly higher on both TCP and TiN than in TNFα‐activated control ECs, indicating their physiological antithrombogenic status, consistent with the findings of *Gojova* et al.^[^ ^62^ ^]^ Thus, both the tested citrate coating and loading time proved to be suitable for effective IONP uptake while ensuring a viable and physiologically functional EC phenotype, as a prerequisite for further experiments.

In the first translationally relevant step, the rotor blades of the original HVAD impeller were statically endothelialized. Based on magnetic flux density, they were categorized into three ROIs, where ROI I (490 mT) and II (400 mT) were significantly higher than the flux density of 200 mT previously applied when using the TiN plate, which was technically limited. Non‐loaded as well as Cit‐IONP‐loaded ECs formed a confluent, viable monolayer with comparable physiological properties. Both endothelialized impellers showed significantly fewer adherent platelets compared to non‐endothelialized ones, confirming the hypothesis of improved hemocompatibility due to their endothelialization. Also independent of IONP internalization and contact with the magnetic TiN surface, both EMLs equally generated the flow stability relevant components collagen type IV, as an essential part of the extracellular matrix,^[^ ^58^ ^]^ and VE‐cadherin, crucial for cell‐cell junctions,^[^ ^71^ ^]^ which are also used as indicators for physiologically appropriate and functioning EC phenotype. Compared to the homogeneous distribution of the non‐loaded EML, the IONP‐loaded ECs have been aligned along the magnetic flux density, which is reflected qualitatively and quantitatively in the results. This is a first indication that the magnetic attraction has an adhesion‐promoting effect on the IONP‐loaded ECs, whereby the IONP‐loaded ECs can ensure the immediately necessary flow stability on rotating impellers until the formation of the basement membrane matrix^[^ ^72^ ^]^ and the intercellular contacts, which are additionally influenced by shear stress,^[^ ^73^ ^]^ can sufficiently take over this task until the support by magnetic attraction is diminished with the successively decreasing IONP concentration due to proliferation or extended application time.

### 4.3 Cellular Magnetic Attraction Provides Confluent Eml Under Dynamic Conditions on Original LVAD Impeller

To successfully evaluate the potential contribution of magnetic attraction to the ability of Cit‐IONP‐loaded ECs to immediately resist clinically relevant shear stresses at the impeller surface, we developed different mock circulation loops. With the first experimental setup FR1, average shear rates of 10.61 Pa were achieved on the endothelialized impeller surface at 1000 rpm, so these EMLs were exposed to almost physiological shear stresses, which are given in the literature as 0.1 to 9.5 Pa.^[^ ^74^ ^]^ Although the total endothelialized surface area was almost unchanged after 24 h of flow application for non‐loaded and Cit‐IONP‐loaded ECs, their distribution showed significantly more Cit‐IONP‐loaded ECs for the highly magnetic regions ROI I and II, which was already the case under static conditions. The same picture was seen in FR2 with 10‐fold increased supraphysiological shear rates, which averaged 124.37 Pa. However, in contrast to the Cit‐IONP‐loaded ECs, the impeller region populated with non‐loaded ECs was significantly reduced after flow application, indicating that the internalized Cit‐IONPs actually contributed to EC retention under shear stress.

Regardless of IONP loading, ECs responded to rotation in both FR1 and FR2 with physiological upregulation of shear stress‐associated genes^[^ ^75^ ^]^ such as Vinculin, thrombomodulin,^[^ ^76^ ^]^ and KLF2,^[^ ^77^ ^]^ which are essential for the antithrombogenic and flow‐resistant phenotype under dynamic conditions and whose upregulation appeared to correlate with the level of shear stress. Vinculin is of particular interest here as it is an intracellular protein that strengthens both cell‐cell junctions and anchoring in the extracellular matrix,^[^ ^78^ ^]^ and whose expression is negatively affected by a variety of different IONP coatings but not by citrate‐coated IONPs, confirming the suitability of our selected coating in addition to its proven biocompatibility.^[^ ^79^ ^]^ Overall, these results also suggest, in line with the literature, that successively increased flow‐adaption protocols may enhance endothelial flow resistance to supraphysiological shear stresses, as successfully demonstrated in previous studies for ECs on other cardiovascular implants, such as heart valves.^[^ ^80^ ^]^ This seems highly relevant with regard to the translational application in the original LVAD, as according to CAD calculations at an LVAD minimum running rate of 1800 rpm, the shear rates were calculated to average 225.24 Pa with a maximum of 5451.28 Pa. Therefore, endothelialized impellers were first exposed to a physiological flow profile in FR1 for 24 h before being analyzed in the original LVAD housing under clinically relevant flow conditions. Again, the positive effect of Ci‐IONP loading on EC adhesion to the impeller was demonstrated, as the area with a remaining confluent monolayer was larger and with a higher cell density than with non‐loaded ECs, as already observed in the FR2 setup.

However, it was found that the strong neodymium magnet, which was temporarily attached to the bottom during assembly, could not always prevent the seeded impeller from briefly touching the top lid of the housing before starting the 1800 rpm, which may have been a reason for the observed, shear stress‐independent cell loss under clinical conditions compared to FR2. From this we concluded that reliable and reproducible endothelialization of magnetically mounted impellers is only effective under rotation, circumventing the cumbersome assembly process and thereby avoiding contact between impeller and housing. This is one reason why we aim to apply our seeding approach to next generation, fully magnetically levitated LVAD systems. Without the need for a hydrodynamic bearing, this no‐contact strategy is much more feasible. We were not only able to successfully apply this seeding strategy in the rotating FR1 setup with physiological shear rates, but were also able to further elucidate the indispensable importance of endothelial IONP loading for effective, clinically relevant endothelialization by magnetic attraction. Only this strategy allowed cell seeding under the challenging conditions on the LVAD impeller. Thus, the number of adherent non‐loaded ECs in all three ROI regions was below the detection limit, while ECs loaded with Cit‐IONPs were able to endothelialize both the ROIs with high and low magnetic flux density, whereby their cell density increased significantly with longer rotation time. This is of great importance for a timely clinical implementation, as the proven advantage of Cit‐IONP‐mediated adhesion enhancement could be used even more effectively, especially for the endothelialization of LVAD models with lower shear stress, such as the Heartmate 3.^[^ ^81^ ^]^ This is because this new generation LVAD, which is currently in clinical use, has another decisive advantage over the HVAD aside from the possible contactless seeding approach mentioned above. The larger bearing gaps of at least 500 µm compared to the HVAD with 22 µm lead to a significantly lower wall shear stress of a maximum of 150 Pa,^[^ ^82^, ^83^ ^]^ a value that was very well tolerated by the ECs in this study, so that the clinically relevant generation of a confluent monolayer appears realistic.

Nevertheless, potential cell detachment and the consequence of accidental distribution of cells or Cit‐IONPs into the bloodstream need to be investigated in more detail within the future mock circulation and in vivo studies. In the current protocol, however, a total amount of roughly 0.048 mg iron per impeller is administered (2 × 10^6^ ECs with an average iron load of about 24 pg per cell). This is by far not as high as the doses already confirmed to be safe in human patients.^[^ ^54^ ^]^

## 5 Conclusion and Outlook

In summary, this study established a biocompatible, valid, and reproducible protocol for the internalization of Cit‐IONPs into hCB‐ECFCs, which enabled the cells to immediately form a confluent, viable EML due to their magnetic interaction with the impeller. This is of tremendous importance for the future clinical scenario, especially in acute LVAD implantations, as the presence of magnetic interaction ensures cell adhesion even under clinically relevant conditions with supraphysiological shear rates, so that the artificial impeller surface directly benefits from the significant improvement of hemocompatibility by the ECs. Moreover, this magnetic attraction provides sufficient time for the ECs to respond to the challenging environment with physiological adaptations, like upregulating and synthesizing shear‐protective mechanisms such as basal lamina matrix formation, cell‐cell and cell‐substrate junction reinforcement or glycocalyx production^[^ ^59^ ^]^ to ensure long‐term adhesion under flow.

The fact that IONP loading enables impeller endothelialization even under rotation has a supporting effect, so that possible cell loss during the initial start‐up can be regenerated by circulating IONP‐loaded ECs during operation. Nevertheless, the applicability and long‐term safety of the reported biohybrid approach need to be investigated in a careful step‐by‐step approach prior to clinical implementation. This will demand separate studies, most importantly testing long‐term monolayer stability and antithrombogenicity, the resilience of ECs under contact with whole blood in a mock circulation as well as systemic implications in future animal experiments. Based on current literature, however, it is also conceivable that a partially worn‐off monolayer exposes signals in vivo that could induce endothelial homing into the monolayer for endogenous EC repair.^[^ ^84^ ^]^ Importantly, IONP‐mediated cell attraction can be easily transferred to other LVAD devices that also have a magnetic drive, such as the Heartmate 3.

Overall, this study, therefore, suggests that the use of Cit‐IONP‐loaded ECs has great potential for the development of a biohybrid LVAD in which the blood‐contacting surfaces can be covered with an immunologically compatible EML^[^ ^31^, ^36^ ^]^ that ensures optimal hemocompatibility, so that the use of anticoagulant drugs, which are associated with sometimes fatal side effects, can be significantly reduced or even omitted, thus significantly improving the quality of life of patients. The translation into a contemporary fully magnetically levitated LVAD system with a more favorable shear stress and operation profile is the long‐term goal.

## Conflict of Interest

The authors declare no conflict of interest.

## Author Contributions

J.L.H. and M.P. contributed equally to this work as co first authors. J.L.H. performed the data collection, statistical analyses, and literature search, and wrote the manuscript. In addition to these points, M.P. drafted and critically revised the manuscript. H.J.G., K.K., J.H., F.H., and M.L. carried out the data collection and statistical analyses. U.K., J.A., and A.R. critically revised the manuscript for important intellectual content and final approval of the version. B.W. was responsible for fundraising, conceptualization, and design of the study, drafting and critical revision of the manuscript for key intellectual content, final approval of the version, and is the corresponding author. All authors are responsible for all aspects of the work and are committed to ensuring that issues relating to the accuracy or integrity of any part of the work are appropriately investigated and resolved. All authors have contributed to the article and approved the submitted version.

## Supporting information



Supporting Information

## Data Availability

The data that support the findings of this study are available from the corresponding author upon reasonable request.
