# Supplementary material for: Citrate‐Coated Iron Oxide Nanoparticles Facilitate Endothelialization of Left Ventricular Assist Device Impeller for Improved Antithrombogenicity
Source: Adv Sci (Weinh). 2024 Dec 20;12(6):2408976. doi: 10.1002/advs.202408976 (PMC11809402; doi:10.1002/advs.202408976)
Supplement: Supplementary file 1 — Supporting Information [file ADVS-12-2408976-s001.docx]

Supporting Information

**Citrate-Coated Iron Oxide Nanoparticles Facilitate Endothelialization of Left Ventricular Assist Device Impeller for Improved Antithrombogenicity**

Jasper L. Haritz, Michael Pflaum, Hans J. Güntner, Katherina Katsirntaki, Jan Hegermann, Felix Hehnen, Michael Lommel, Ulrich Kertzscher, Jutta Arens, Axel Haverich, Arjang Ruhparwar, Bettina Wiegmann*

Supplementary Materials and Methods:

Table S1 - Primers used for gene expression analysis (in 2.4.4. and 2.5.3.)

| Gene | Forward 5´-3´ | Reverse 5´-3´ | NCBI Reference |
| --- | --- | --- | --- |
| ELAM | ATC CAG CCA ATG GGT TCG TG | GAA GGC TCT GGG CTC CCA TT | [NM_000450.2](http://www.ncbi.nlm.nih.gov/entrez/viewer.fcgi?db=nucleotide&id=187960041) |
| VCAM-1 | GGC GCC TAT ACC ATC CGA AA | GAG CAC GAG AAG CTC AGG AGA A | [NM_001078.3](http://www.ncbi.nlm.nih.gov/entrez/viewer.fcgi?db=nucleotide&id=315434269) |
| ICAM-1 | CTA CCT CTG TCG GGC CAG GA | AGG CCT GCA GTG CCC ATT AT | [NM_000201.2](http://www.ncbi.nlm.nih.gov/entrez/viewer.fcgi?db=nucleotide&id=167466197) |
| TM | GCC CAT GGG AGC TGG TTA GA | GGC CTG ACT TGG CCT GCT AC | [NM_000361.2](http://www.ncbi.nlm.nih.gov/entrez/viewer.fcgi?db=nucleotide&id=40288292) |
| Tissuefactor | CCC GAA CAG TTA ACC GGA AGA | GGA GTT CTC CTT CCA GCT CTG C | [NM_001993.4](http://www.ncbi.nlm.nih.gov/entrez/viewer.fcgi?db=nucleotide&id=296010910) |
| KLF2 | CGC ACA CAG GTG AGA AGC CC | GGG CGT CCC GGC TAC ATG T | [NM_016270.2](http://www.ncbi.nlm.nih.gov/entrez/viewer.fcgi?db=nucleotide&id=49574523) |
| Vinculin | CAG ACC TTG AAC AAC TCC GAC TAA | CAA CAC CTA TAC CCA CCT CAG C | NM_014000.3 |

Antibodies used for Immunofluorescence (in 2.5.1)

Primary antibodies

- Polyclonal rabbit anti-human VE-cadherin intercellular junction marker (Abcam, REF# ab33168), 5 µg / ml.
- Monoclonal mouse anti-human collagen IV clone CIV 22 (Dako, REF# M0785), 0.66 µg /ml.

Secondary antibodies

- Cy3-conjugated AffiniPure donkey anti-mouse IgG (Jackson ImmunoReseacrh, REF# 715-165-151), 2 µg / ml.
- Alexa Fluor 488-conjugated donkey anti-rabbit IgG (Jackson ImmunoResearch, REF# 711-546-152), 0.3 µg / ml.
- Hoechst 33342, 2 µg / ml.

Assessment of IONP-induced mitochondrial damage

As an indicator for the possible onset of ferroptosis, EC monolayers were loaded with Cit-IONPs at high and low dose for 24 h and forwarded to flow cytometry analysis, after staining for functioning mitochondria. Therefore, loaded and non-loaded ECs were detached from the cavities of 6 well plates using Trypsin /EDTA, centrifuged and resuspended in DMEM/10% FBS medium containing 500 nM Tetramethylrhodamin-methylester (TMRM, Thermo Fisher Scientific) and 500 nM MitoTracker Deep Red FM (TMRM, Thermo Fisher Scientific). After 30 min incubation at 37°C, stained cells were washed with PBS, centrifuged and resuspended in MACSQuant Running buffer (Miltenyi). Using the MACSQuant X (Miltenyi), 2 x 10^4^ events were recorded and analyzed using the FlowLogic software version 700.0A (Inivai Technologies, Mentone, Australia). After excluding the doublets events in the main population, the mean fluorescence intensity and percentage of TMRM positive and MitoTracker Deep Red positive events were used to analyze the impact of the IONP loading dose. The non-loaded ECs served as non-affected reference, while non-loaded ECs incubated with 70% Ethanol for few seconds were used as negative control for mitochondria without membrane potential.

In order to investigate changes to the mitochondrial morphology indicative of detrimental processes during ferroptosis, TEM images of ECs treated with a low dose of Cit-IONPs for 24 h, obtained in 2.4.1. were evaluated in parallel to the FACS analysis.

Figure S1 a)

Figure S1a: Assessment of functioning mitochondria in Cit-IONP-loaded ECs for 24h. left: Quantification of MitoTracker Deep Red FM positive cells with a TMRM signal intensity above the threshold.Right: Mean fluorescence intensity of TMRM in MitroTracker Deep Red positive cells. Data was compared using the one-way ANOVA test, where all groups were tested against the non-loaded ECs using Bonferroni’s multiple planned comparison test. **: p<0.01, ****: indicated p<0.0001, (n=3).

Figure S1b)


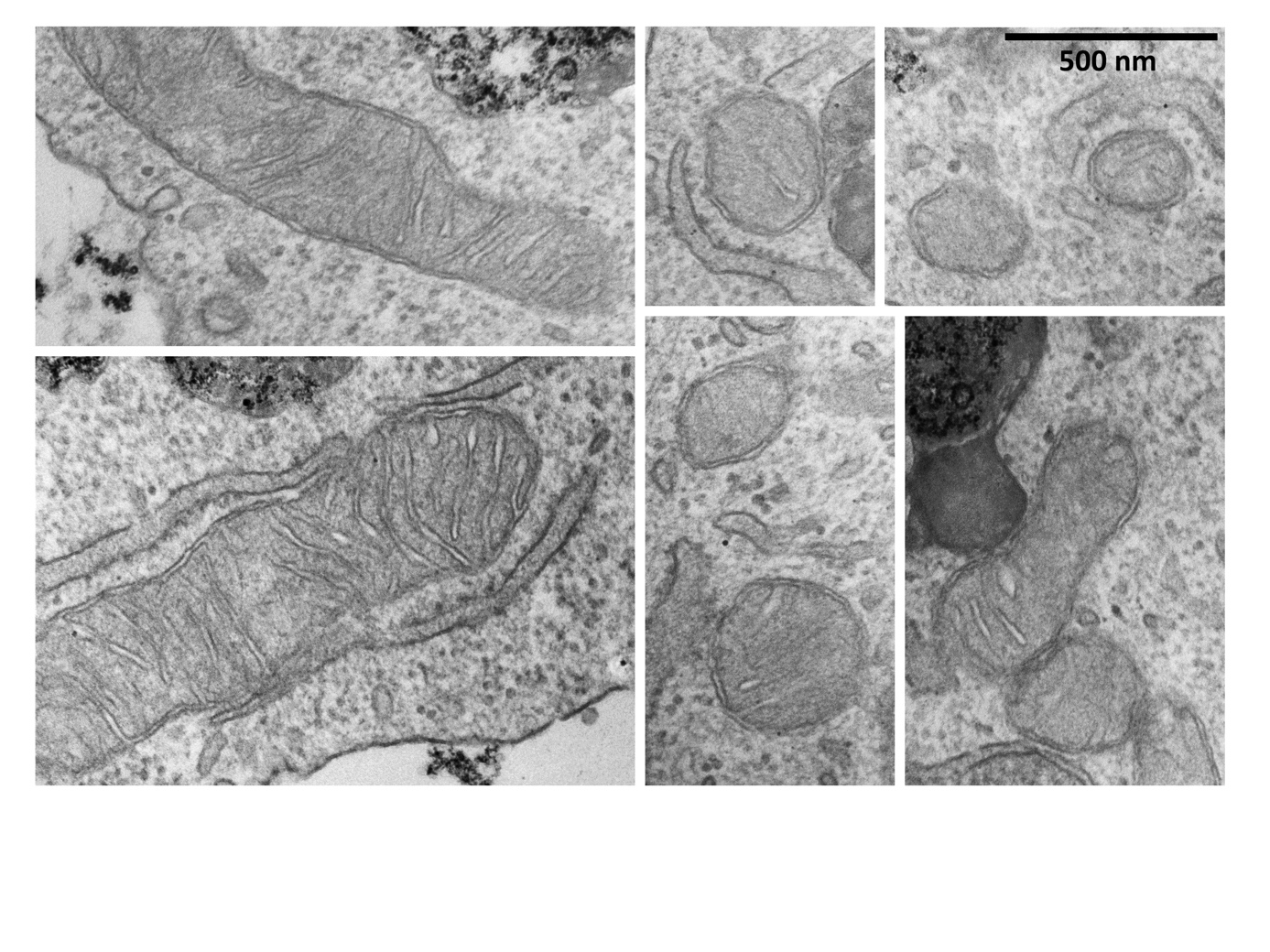


Figure S1b: TEM images of mitochondria in 24h-loaded ECs with low dose Cit-IONP. No morphological abnormalities, indicative of ferroptosis or lipid peroxidation, could be observed.


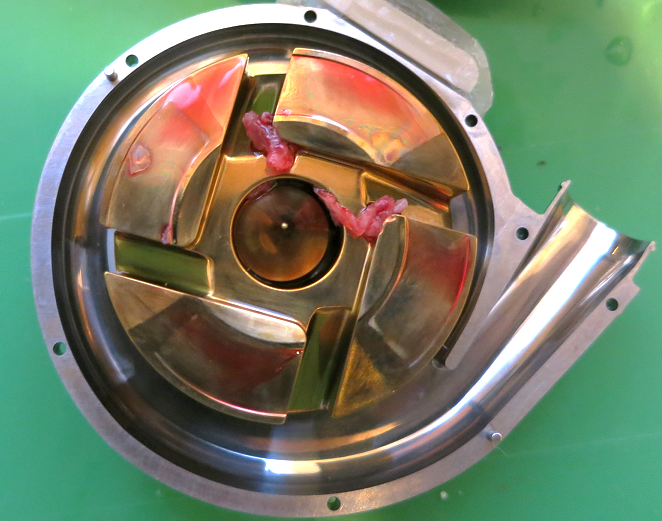


**Figure S2 Opened HVAD after explantation, with thrombus formation on impeller surface.**

**Table S2: Results of the gene expression analysis of ECs and Cit-IONP loaded EC seeded on TCP (control) or TiN. Results are given as mean 2^-^**^Δ^**^Ct^ with standard deviation.**

|  | control | | control / TiN | | + IONPs | | + IONPs / TiN | | + TNFa | |
| --- | --- | --- | --- | --- | --- | --- | --- | --- | --- | --- |
| Gene name | mean | S.D. | mean | S.D. | mean | S.D. | mean | S.D. | mean | S.D. |
| ELAM | 0,0002332 | ±0,00001976 | 0,0001242 | ±0,00003738 | 0,00004328 | ±0,0000119 | 0,00005014 | ±0,00001221 | 0,329 | ±0,01525 |
| VCAM-1 | 0,00003123 | ±0,000006046 | 0,00002861 | ±0,000003456 | 0,000003677 | ±0,000001105 | 0,000004032 | ±0,000003276 | 0,03684 | ±0,00273 |
| ICAM-1 | 0,004982 | ±0,0004729 | 0,003324 | ±0,0002238 | 0,004949 | ±0,001974 | 0,003607 | ±0,0002185 | 0,4106 | ±0,01645 |
| TM | 0,002344 | ±0,0004925 | 0,003594 | ±0,0008309 | 0,001711 | ±0,0007946 | 0,001908 | ±0,0002848 | 0,0002751 | ±0,0000195 |
| Tissuefactor | 0,001334 | ±0,0000397 | 0,001322 | ±0,00004723 | 0,00126 | ±0,0004511 | 0,0005714 | ±0,00009773 | 0,002538 | ±0,0001372 |

**Table S3 – Results of the gene expression analysis of ECs and Cit-IONP loaded EC seeded on the impeller under static conditons and set under rotation at 1000 rpm in FR1 or FR2. Results are given as mean 2^-^**^Δ^**^Ct^ with standard deviation.**

|  | ECs / stat | | ECs / 1000 rpm | | ECs + IONPs / stat | | ECs + IONPs / 1000 rpm | |
| --- | --- | --- | --- | --- | --- | --- | --- | --- |
| FR1 | mean | S.D. | mean | S.D. | mean | S.D. | mean | S.D. |
| KLF2 | 0,0525 | ±0,04008 | 0,2657 | ±0,11 | 0,02014 | ±0,00265 | 0,214 | ±0,07752 |
| TM | 0,005159 | ±0,0012 | 0,04814 | ±0,0002546 | 0,005585 | ±0,001507 | 0,04137 | ±0,01386 |
| Vinculin | 0,0002581 | ±0,00002264 | 0,0003485 | ±0,00004215 | 0,000102 | ±0,00002399 | 0,0004063 | ±0,00008448 |
|  |  |  |  |  |  |  |  |  |
| FR2 |  |  |  |  |  |  |  |  |
| KLF2 | 0,04018 | ±0,01118 | 0,4022 | ±0,08263 | 0,04263 | ±0,03045 | 0,3854 | ±0,2225 |
| TM | 0,00978 | ±0,005985 | 0,041 | ±0,009907 | 0,007185 | ±0,003015 | 0,05101 | ±0,01016 |
| Vinculin | 0,0001699 | ±0,00005484 | 0,0006099 | ±0,0001398 | 0,0001916 | ±0,00007162 | 0,0006451 | ±0,0002353 |


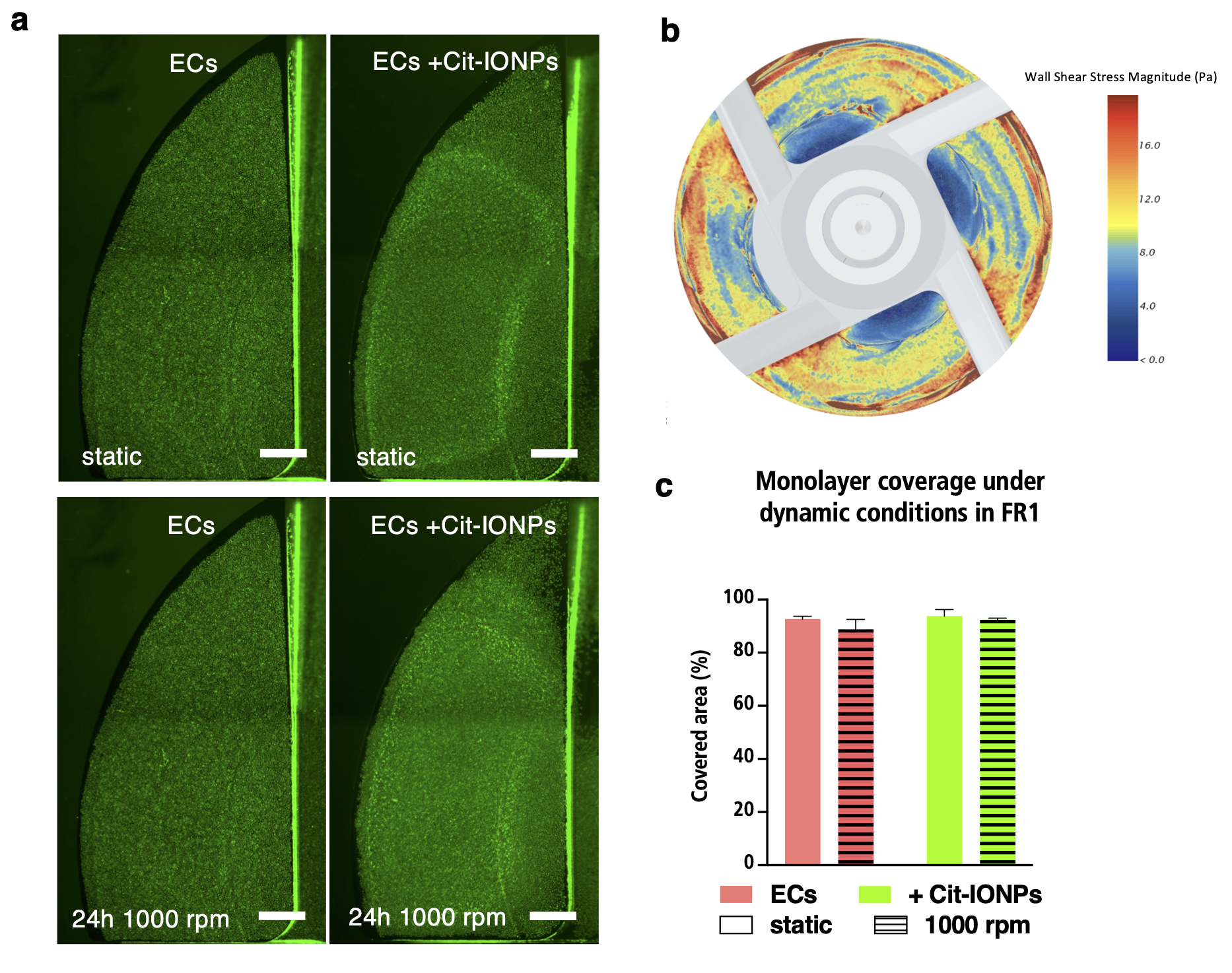


**Figure S3: Quantification of EC monolayer coverage on the impeller under static and after 24 h at 1000 rpm in flow reactor FR1. (a) Fluorescence microscopy of calcein (green) stained monolayers with and without Cit-IONP and under static (left column) and after 24 h at 1000 rpm (right column); Scalebar: 1 mm. (b) Heat map of shear stress distribution on the impeller surface in FR1 at 1000 rpm. (c) Quantification of surface area on impeller surface with intact and confluent EC monolayer.**


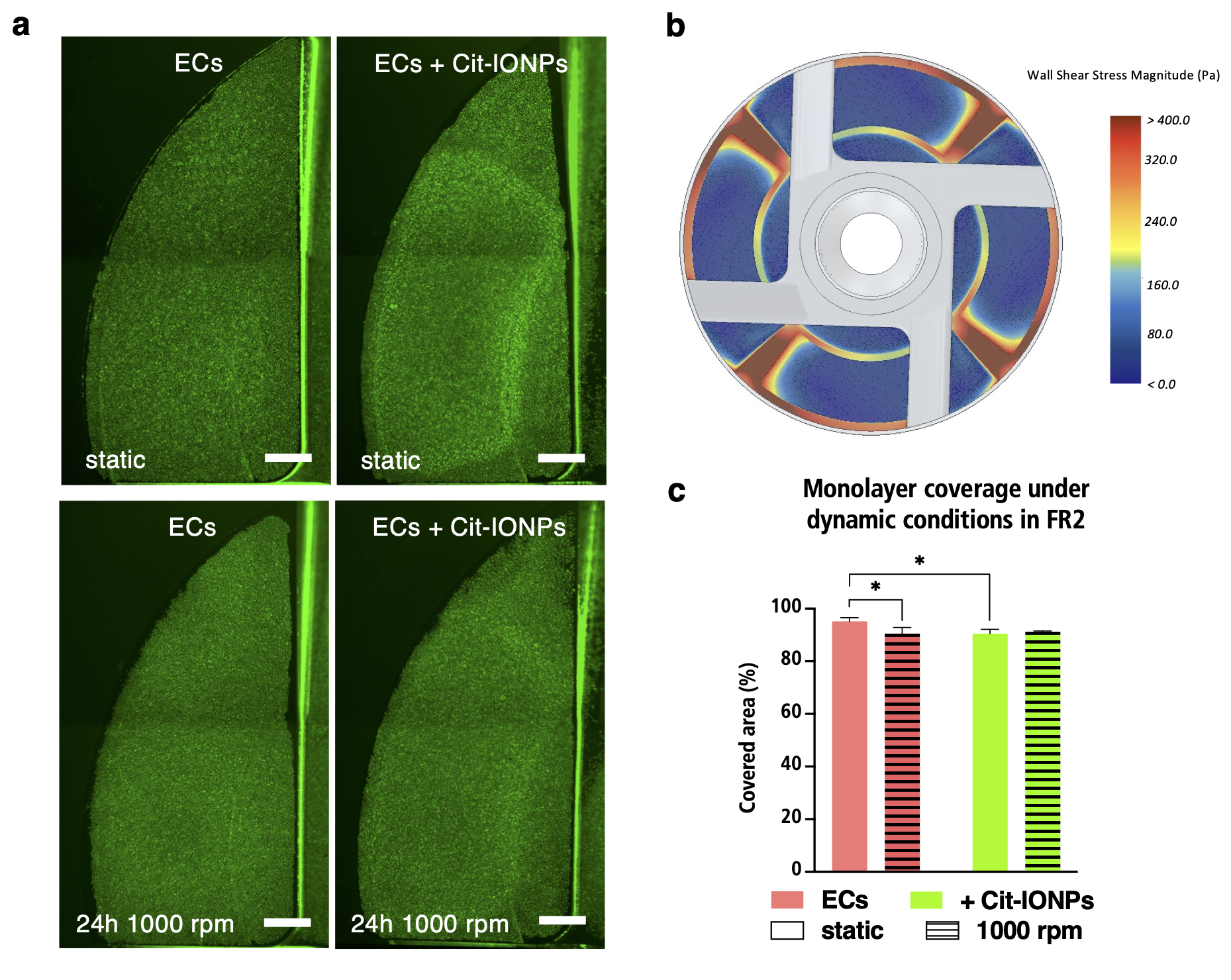


**Figure S4 Quantification of EC monolayer coverage on the impeller under static and after 24 h at 1000 rpm in flow reactor FR2. (a) Fluorescence microscopy of calcein (green) stained monolayers with and without Cit-IONP and under static (left column) and after 24 h at 1000 rpm (right column); Scalebar: 1 mm. (b) Heat map of shear stress distribution on the impeller surface in FR2 at 1000 rpm. (c) Quantification of surface area on impeller surface with intact and confluent EC monolayer.**


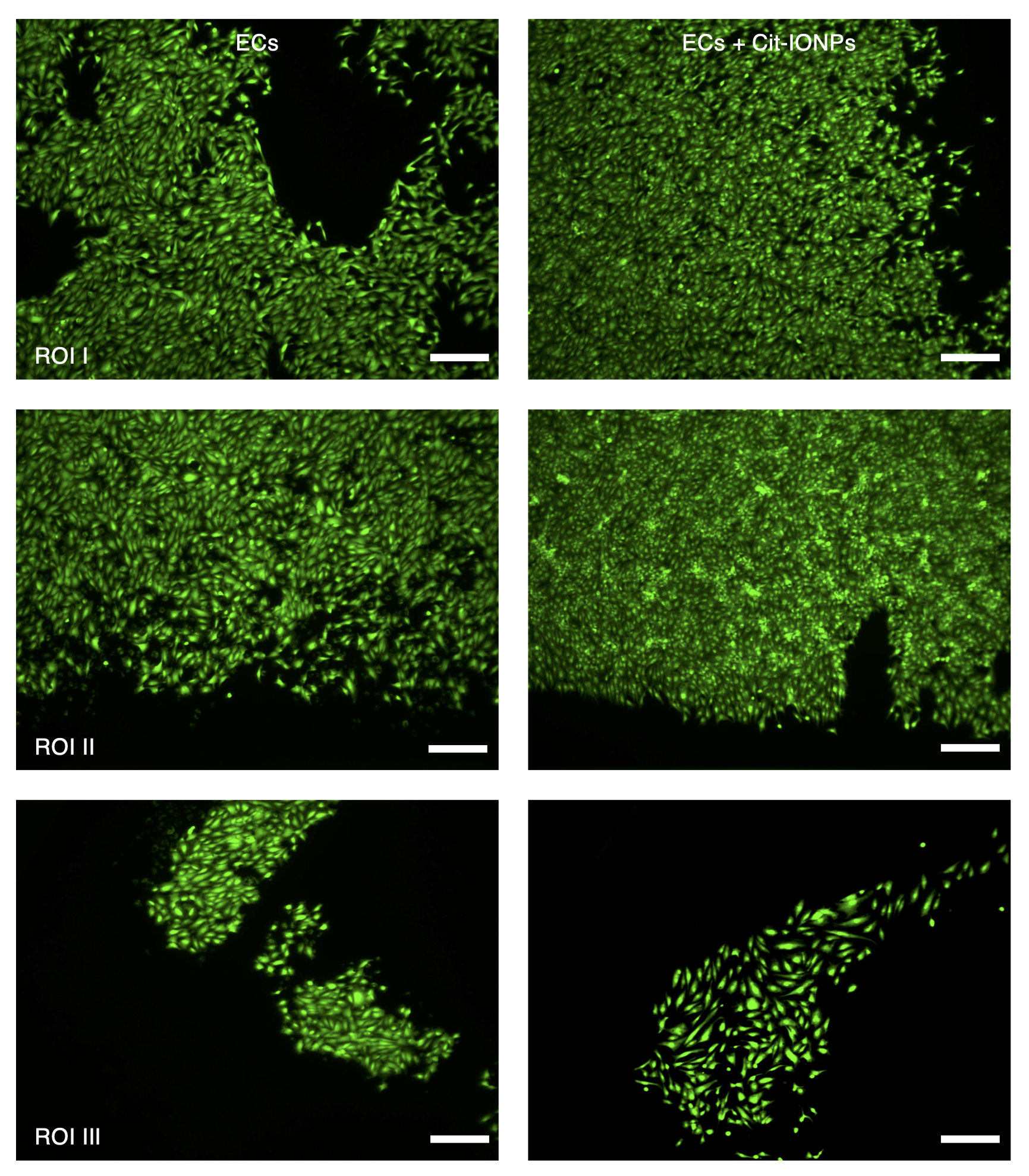


**Figure S5: Fluorescence microscopy of calcein (green) stained non-loaded ECs (left column) and Cit-IONP-ECs (right column) following static and dynamic preincubation and operation at 1800 rpm for 1 h within the original HVAD housing. Each row contains representative images from another ROI (see Figure 4a); Scalebar: 250 µm.**
